# The Relationship Between Polygenic Risk of Depression and Regional Brain Atrophy in Older Adults

**DOI:** 10.1002/gps.70245

**Published:** 2026-08-16

**Authors:** Lindsey I. Sinclair, Clive G. Ballard, David A. Bennett, Jennifer C. Palmer, For the Alzheimer's Disease Neuroimaging Initiative, Sarah E. Medland

**Affiliations:** ^1^ Cerebrovascular and Dementia Research Group Bristol Medical School University of Bristol Bristol UK; ^2^ Department of Psychiatry Faculty of Medicine University of Southampton Southampton UK; ^3^ Medical School University of Exeter Exeter UK; ^4^ Rush Alzheimer's Disease Center Rush University Medical Center Chicago Illinois USA; ^5^ Department of Neurological Sciences Rush University Medical Center Chicago Illinois USA; ^6^ Bristol Medical School NIHR Bristol Evidence Synthesis Group University of Bristol Bristol UK; ^7^ QIMR Berghofer Brisbane Australia

**Keywords:** dementia, depression, depressive disorder, polygenic risk

## Abstract

**Background:**

Depression has been identified as a risk factor for the development of dementia, but the relationship between the two conditions is complex. If the mechanism by which depression increased the risk of dementia was better understood then new treatment targets could be identified. Previous studies have found evidence of reduced hippocampal volume in depressed patients, but because depression may also be an early sign of dementia, these studies may have inadvertently included individuals in the early stages of dementia. We used genetic risk of depression as our exposure and examined whether older adults with the highest genetic risk of depression had reduced regional brain volumes or greater longitudinal regional atrophy.

**Methods:**

We used clinical, genetic and neuroimaging data from the ROSMAP and ADNI studies, which are both older adult focused longitudinal cohort studies with derived regional brain volumes a subset of available from MRI scans. Polygenic scores for depression were generated in PLINK from genotyped data imputed using 1000 genomes Phase 3 v5 using the clumping and thresholding method, based on the Howard et al., 2019 GWAS of depression. In ADNI *n* = 473 at baseline and 342 at 12 months, in ROSMAP *n* = 187 at baseline and *n* = 113 at 12 months. In a secondary analysis we excluded those with dementia at baseline. Our primary outcome was the effect of PRS‐D on hippocampal volume in both studies using linear regression. The results from the 2 studies were then meta‐analysed.

**Results:**

We did not find any evidence of an effect of polygenic risk for depression on regional brain volume changes at baseline or after 12 months, either in ROSMAP or ADNI individually or in the meta‐analysis. This finding was not affected by excluding individuals with evidence of dementia at baseline in ADNI or in ROSMAP those with AD at post‐mortem examination.

**Conclusions:**

We found no strong evidence of an effect of polygenic risk of depression on regional brain volumes in older adults either at baseline or after 12 months. Our work was limited to genetic risk of depression and further research is required to investigate the complex relationship between depression and dementia.

## Key Points

- We used data from two large cohorts focused on older adults, ROSMAP and ADNI, to examine the effect of genetic risk of depression on regional brain volumes.
- We did not find any evidence of an effect of polygenic risk for depression on regional brain volume at baseline in either study.
- We did not find any evidence of an effect of polygenic risk for depression on regional brain volume changes after 12 months, either in ROSMAP or ADNI individually or in the meta‐analysis.
- Our work was limited to genetic risk of depression and further research is required to investigate the complex relationship between depression and dementia.

## 1 Introduction

There is a complex relationship between depression and dementia [1]. Depression in mid to later life is a risk factor for the later development of dementia, but the mechanism of this is unclear. For some individuals, depression can also be an early AD symptom [1]. Depression is also more common in people living with dementia, including AD, than in older adults without dementia [1, 2]. It is unclear whether the way depression is treated, or whether it is treated at all affects this increase in risk. One previous study has shown that treatment of later life depression may reduce the risk of subsequent dementia in some, but not all patients [3].

In our work to date we have shown, using post‐mortem human brain tissue [4] that later life depression is not an early sign of AD, nor does it seem to be related to reduced brain perfusion. Specifically, there was no evidence of increased Aß, tau, a‐synuclein, chronic hypoperfusion or neuroinflammation compared to controls. Using data from large cohort studies we have also shown that individuals with mid to later life depression perform worse in some (but not all) cognitive tests even at 15‐year follow‐up, but that this does not seem to be linked to neuroinflammation [5, 6]. This persistent alteration of cognitive function suggests that depression may lead to lasting changes in brain function and/or morphology.

Better understanding of the mechanisms by which depression increases the risk of AD could lead to identification of new targets for treatment development, including for AD prevention. The hippocampus is one of the earliest brain areas affected by atrophy in AD [7, 8]. Hippocampal atrophy in older adults with depression has been shown in some but not all studies [9, 10, 11, 12, 13]. In a meta‐analysis that was not restricted to older adults, patients with depression had lower hippocampal volumes, particularly those with recurrent depression, which could reduce cognitive reserve [14]. A cross‐sectional analysis found that adults aged under 75 with depression appear to have greater predicted “brain aging” than controls [15]. Because depression can also be an early AD symptom [1] such studies in older adults may include individuals who are already in the early stages of dementia [10]. Using genetic risk of depression as the exposure reduces the potential for confounding. The longitudinal effects of genetic risk of depression on regional brain volume in older adults are unknown. Previous studies have been cross‐sectional and/or studied younger adults [16, 17, 18].

We aimed to study the effect of genetic risk of depression on longitudinal brain atrophy in older adults. Our hypothesis was that polygenic risk for depression in individuals aged > 50 increases the rate of regional brain atrophy over time in brain areas known to be involved in the control of mood and/or affected in Alzheimer's disease.

## 2 Methods

We used clinical, genetic and neuroimaging data from the ROSMAP and ADNI studies, which are both older adult focused longitudinal cohort studies with derived regional brain volumes available from MRI scans.

### 2.1 ROSMAP

The ROSMAP cohort combines the Religious Orders Study and Rush Memory and Aging Project. Both studies were designed to study cognitive health in aging. Participants agreed to annual clinical evaluation and brain donation at the time of death [19]. Both studies recruited individuals without known dementia. The Religious Orders Study recruited Catholic clergy from about 40 religious communities across the US (including one small group of Latinx communities and three primarily African American orders) [20] and its participants had an unusually high prior educational attainment. MAP is much more representative of the wider US older adult population [19]. MAP participants were recruited from retirement communities in Illinois, retirement homes, churches and social service agencies, and individual homes. All evaluations in both studies were home visits, which again aimed to increase the representativeness of this study population, reduce the healthy volunteer effect, and maximize clinical data proximate to death [21]. There is a large common core of data identical at the item level in both studies which allows for efficient joint analyses. Both studies were approved by an Institutional Review Board of Rush University Medical Center. All participants signed informed and repository consents and an Anatomic Gift Act.

In ROSMAP clinicians indicated whether depression meeting DSM‐III‐R criteria was present or not present at each visit [20]. Depressive symptoms were assessed using the CESD [22].

**FIGURE 1:**
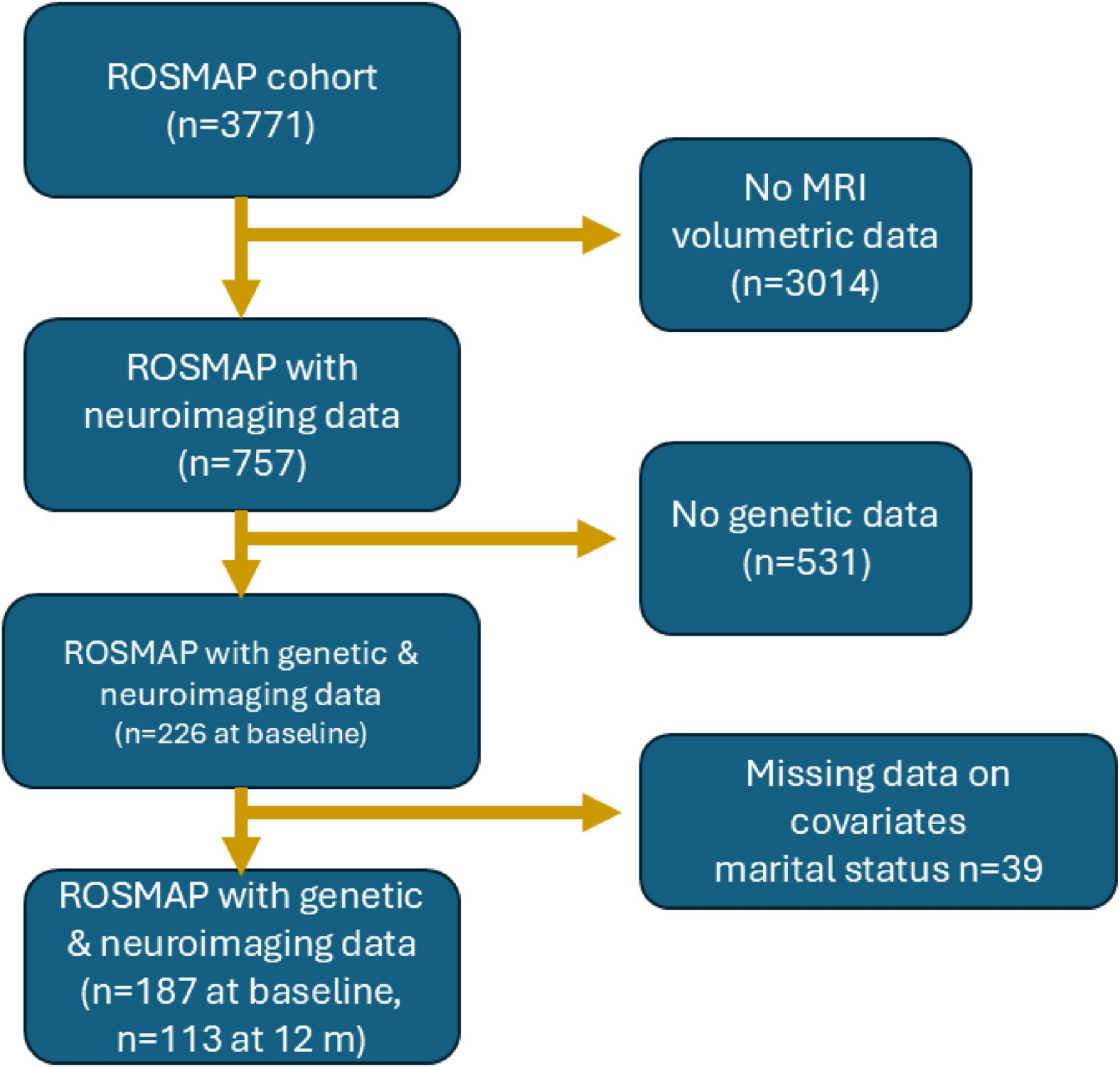
Flow diagram for the ROSMAP sample used in this study.

Genetic data in ROSMAP was collected in 2 phases: ROSMAP1 (*n* = 1695, Affymetrix 6.0) and ROSMAP2 (*n* = 544, Human Omni express). As there was sufficient overlap between the SNPs directly genotyped on each chip, population stratification was performed using the unimputed data [23]. After data cleaning (see Figure 1) there were 757 individuals who had had at least one MRI scan, from which regional volumetric data had been generated using freesurfer and multi‐atlas segmentation then corrected for total intracranial volume [24]. Once merged there were 226 individuals with genetic data and MRI data available, but of those 113 individuals with data from more than one MRI scan. In ROSMAP post‐mortem neuropathological assessments had been performed in line with NACC recommendations, wherever possible, for individuals who had passed away [25]. In brief sections were stained with modified Bielschowsky for neuritic plaques and neurofibrillary tangles. These were summarized by CERAD, Braak stage and NIA‐Reagan criteria as previously described [26, 27]. The evaluation included an assessment of nigral, limbic and neocortical Lewy bodies, hippocampal sclerosis, TDP‐43, and measures of micro‐ and macro‐vascular disease as previously described [28].

### 2.2 ADNI

**FIGURE 2:**
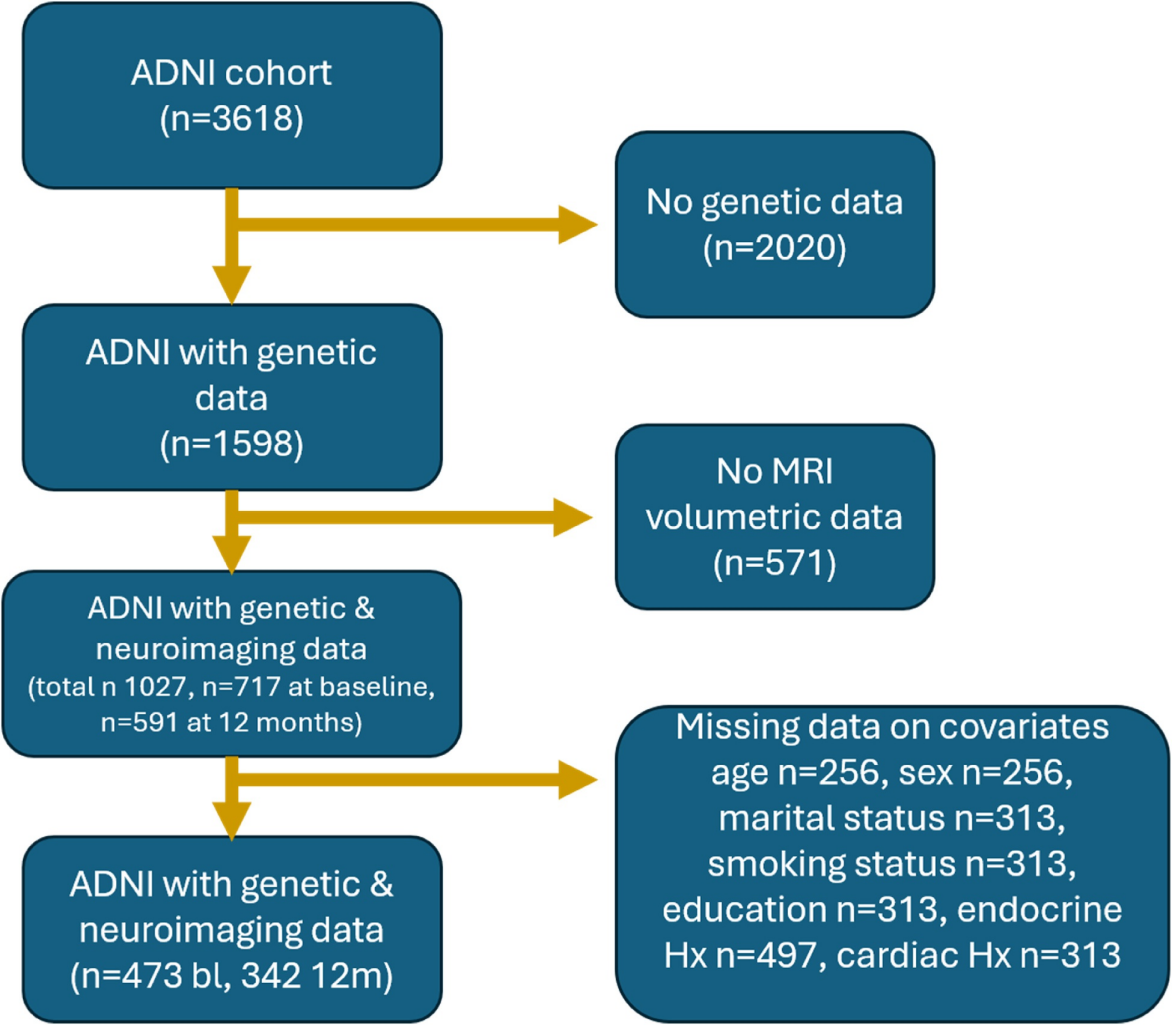
Flowchart for the ADNI sample used in this study.

ADNI (https://adni.loni.usc.edu) was established in 2003 and was carried out in phases; ADNI1 in 2004; ADNI‐GO in 2009; ADNI 2 in 2011; and ADNI3 in 2016 [29]. Existing participants were included in the recruitment for each successive phase. The data collected in each phase differs slightly. Depression either at entry to the study or in the 2 years preceding study entry was an exclusion criterion, meaning that very few participants had significant depressive symptoms at baseline. Depressive symptoms were measured using the Geriatric Depression Scale (GDS) and the NPI/NPI‐Q [29, 30, 31]. The final dataset was downloaded in March 2024. Regional brain atrophy data used in this study was derived by Ledig et al. and parcellated using the Neuromorphometrics (NMM) brain atlases [32], which had been corrected for total brain size. The data cleaning process to result in the final number of participants is illustrated in Figure 2.

Genetic data in ADNI was collected in 4 phases: ADNI1 (*n* = 757), ADNI2 (*n* = 361), ADNI3 (*n* = 327) and ADNI3 set 2 (*n* = 327). The Illumina Infinium global screening array was used in ADNI3, the Human omni express in ADNI2, and the Human610‐quad bead chip (Illumina) in ADNI1 [33]. As there was insufficient overlap in the SNPs directly genotyped on each chip, population stratification was performed using imputed genetic data [23].

### 2.3 Ethical Approval

Both ROS and MAP were approved by an Institutional Review Board of Rush University Medical Center and all participants signed an Anatomic Gift Act as well as repository and informed consents [19]. ADNI obtained ethical approval from the Institutional Review Boards of all of the participating institutions [34]. Both studies obtained informed consent from participants at the point of study entry, including for secondary use of their data. As this study's use of ADNI and ROSMAP data fell into the category of secondary analysis of anonymised data, under UK law, no separate ethical approval was required for these analyses.

### 2.4 Polygenic Methods

Polygenic risk scores for depression (PRS‐D) have been shown to explain at least 3% of the liability to depression [35] which compares well to other risk factors in behavioral neuroscience [36]. We used clumping and thresholding to calculate PRS‐D using the Howard et al. depression GWAS [37, 38]. Unimputed genetic data from both studies was cleaned in PLINK 1.9 (using thresholds of individual missingness 0.02, genotype missingness of 0.02, minor allele frequency of 0.05, HWE 10^6^, relatedness of 0.2) and then imputed using the Michigan Imputation server v1 with 1000 Genomes Phase 3 v5 as the reference panel [39]. We then used the vcf files from the Michigan Imputation server in PLINK 2.0 to generate the clumping data. For the clumping a MAF threshold of > 0.005, an imputation quality score of > 0.6, an *r* ^2^ of 0.1 and a genetic distance of 10,000 were used. Multiple thresholds were examined for the threshold *p* value (0 0.000001, 0.001, 0.01, 0.20, 0.20, 0.50, 0.70 and 1.0). We chose *p* < 0.1 as the threshold that explained the most variance in the maximum score per individual on the CESD (ROSMAP) and the GDS (ADNI) as an outcome measure, with age, sex and the first 4 population stratification principal components as co‐variates.

### 2.5 Statistical Analysis and Outcomes

Genetic data was cleaned in PLINK 1.9 and 2.0 and the PRS scores were generated using PLINK 1.9. Data analysis was performed in STATA v15, apart from the meta‐analysis and number of independent brain regions analysis which was performed using meta/metafor and matspd in R [40]. Matrix spectral decomposition (matSpD) uses eigenvalues from spectral decomposition to estimate the number of independent variables in a correlation matrix [41]. Our primary outcome was the effect of PRS‐D on hippocampal volume at baseline in both studies using linear regression. Age at baseline, sex, years of education, marital status, hypertensive status, diabetic status, smoking status at baseline, the heaviest lifetime alcohol consumption and the first 4 principal components from the population stratification analysis were included as co‐variates [1, 42, 43, 44, 45]. Because of the ADNI recruitment methods this study included a far greater number of individuals at baseline with dementia or MCI than ROSMAP. In ADNI therefore clinically assessed cognitive status at baseline, which incorporated performance on validated memory tests, was included as a covariate.

Secondary outcomes included the effect of PRS‐D on the volume of other brain regions and the effect of PRS‐D on atrophy over time. In an a priori secondary analysis, individuals with dementia were excluded from the analysis in order to examine the effect of PRS‐D on brain volumes in individuals without dementia. Bilateral brain volumes were calculated as the average of left and right volumes for each area. The time interval between each MRI scan was calculated in years and brain atrophy over time for each region was calculated as (volume in scan 2—volume in scan 1)/length of time between scans to give an atrophy rate per year.

Based on our previous work and published studies we estimated that 400 individuals would be required in this study, but there was insufficient data to perform a formal power calculation [15, 18, 46]. Assuming an *α* of 0.05 and a desired β(power) of 80%, total *n* = 55 would be required for an effect size of 0.15, *n* = 81 for an effect size of 0.10 and *n* = 395 for an effect size of 0.02.

## 3 Results

### 3.1 ROSMAP

After data cleaning and merging there were 187 individuals in ROSMAP with MRI regional volumetric and polygenic risk score data available. There were 113 individuals who had had 2 or more MRI scans with regional volumetric data available. As shown in Table 1 the mean age at baseline of individuals with at least one scan was 76.7. The majority were female and had never smoked. Only 31 individuals had been assessed by a clinician as meeting DSM‐III depression criteria and 31 were taking antidepressants at baseline. Post‐mortem neuropathological data was available for 100 of these individuals.

**TABLE 1:** Demographic and clinical information for the ROSMAP sample used in this study.

Variable	All (*n* = 187)	No AD at post‐mortem (*n* = 133)	Second MRI scan available for atrophy analysis (*n* = 113)
Age at baseline (y)	76.7 (7.1)	75.3 (7.1)	75.2 (6.8)
Sex			
Female	141	104	85
Male	46	29	28
Years of education (y)	15.6 (3.0)	15.5 (3.1)	15.6 (2.7)
Marital status at baseline			
Never married	13	12	6
Married	90	66	58
Widowed	59	36	32
Divorced	25	19	17
Smoking status at baseline			
Never smoked	100	65	55
Ex smoker	84	66	57
Current smoker	3	2	1
Hypertension			
No	56	42	34
Yes	131	91	79
Diabetes			
No	141	102	83
Yes	46	31	30
Episodes of DSM‐III depression during study			
No	149	107	88
Once	31	23	21
More than once	7	3	4
Maximum CESD score during study	3.1 (2.3)	3.1 (2.2)	3.0 (2.3)
Clinically significant previous head injury at baseline			
No	176	128	103
Yes	11	5	10
Heaviest ever EtOH consumption at baseline			
< 1 drink/month	76	51	44
1–3 drinks/month	19	14	6
1 drink/week	9	4	2
2–4 drinks/week	16	12	13
5–6 drinks/week	9	5	5
1 drink/day	34	29	28
2–3 drinks/day	17	14	10
≥ 4 drinks/day	7	4	5
Clinical diagnosis of Parkinson's disease			
Highly probable	5	1	4
Probable	14	9	8
Possible	48	30	28
Not present	120	93	73
Antidepressant treatment in last 2 weeks at baseline			
No	156	111	95
Yes	31	22	18
Clinical diagnosis of stroke			
Highly probable	8	6	6
Probable	24	17	15
Possible	14	11	9
Not present	141	99	83
Mean PRS‐D	0.002 (0.009)	0.002 (0.0009)	0.002 (0.0009)
Cognitive status at baseline			
Normal cognition	159	112	102
MCI	28	21	11
Dementia	0	0	0
Final consensus cognitive diagnosis after last visit			
Normal cognition	43	29	26
MCI	18	7	8
Dementia	42	13	20
Met AD NCI‐Reagan criteria at post‐mortem			
Yes	54	0	16
No	28	24	24

### 3.2 ADNI

After data cleaning and merging there were 473 individuals in ADNI with baseline MRI regional volumetric and polygenic risk score data available. There were 342 individuals who had had 2 or more MRI scans with regional volumetric data available. Men and smokers were better represented in ADNI (see Table 2).

**TABLE 2:** Demographic and clinical information for the ADNI sample used in this study.

Variable	Baseline volumetric analysis	12 month atrophy analysis	Baseline volumetric analysis with no dementia at baseline (*n* = 317)
	(*n* = 473)	(*n* = 342)	
Age at baseline (y)	75.0 (6.6)	75.1 (6.7)	74.9 (6.2)
Sex			
Female	211	157	135
Male	262	185	182
Years of education (y)	15.5 (3.0)	15.5 (3.0)	16.0 (2.8)
Marital status at baseline			
Never married	14	9	10
Married	373	273	248
Widowed	58	41	40
Divorced	28	19	19
Smoking status at baseline			
Never smoked	284	204	190
Ever smoked	189	138	127
Self‐reported cardiovascular disease			
No	127	92	82
Yes	346	250	235
Self‐reported endocrine disease			
No	285	211	196
Yes	188	131	121
Self‐reported neurological condition			
No	320	238	208
Yes	153	104	109
Maximum GDS score	2.8 (2.5)	2.7 (2.4)	2.9 (2.7)
Number of time scored GDS > 8 during study follow‐up			
0	435	318	285
1	29	21	23
2	5	1	5
≥ 3	4	2	4
History of EtOH abuse			
No	450	324	303
Yes	23	18	14
Antidepressant treatment in last 2 weeks at baseline			
No	470	339	316
Yes	3	3	1
Mean PRS‐D	0.002 (0.009)	0.002 (0.009)	0.002 (0.009)
Cognitive status at baseline			
Normal cognition	153	124	153
MCI	164	103	164
Dementia	156	115	0
Number of APOE ε4 alleles			
0	221	163	172
1	202	141	120
2	50	38	25

### 3.3 Baseline Volumetric Analysis

There was no convincing evidence of an effect of PRS‐D on regional brain volumes at baseline in ROSMAP, including in the hippocampus and other regions of the temporal lobe, as shown in Supporting Information S1: Table s1. Excluding individuals with AD at post‐mortem there was no convincing evidence of an effect of PRS‐D on baseline regional brain volumes, as shown in Supporting Information S1: Table s2. Using matspd there were 40 independent brain regions meaning that a *p* value threshold of 0.00128 would be required for statistical significance.

For the ADNI baseline analysis the number of independent regions was 34, with a revised *p* value threshold of 0.0015. In the baseline analysis none of the regions had a *p* value that met this threshold, although there was a trend toward the right precentral and left cuneus being reduced in size (see Supporting Information S1: Table s4). When individuals with dementia at baseline were excluded there was no evidence of an effect of PRS‐D in the baseline analysis (see Supporting Information S1: Table s6).

### 3.4 Regional Atrophy Analysis

There was data from 113 individuals for the ROSMAP atrophy analysis, 24 of whom had AD at post‐mortem examination. The results of this analysis are shown in Supporting Information S1: Table s3. Using matspd there were 33 independent brain regions, resulting in a *p* value threshold of 0.00155 for statistical significance. These results were underpowered but were combined with the ADNI atrophy results in a random effects meta‐analysis. A secondary analysis of atrophy in those without AD at post‐mortem was therefore not performed as it would have been extremely underpowered.

Using matspd the number of independent regions in the ADNI atrophy analysis was 55, meaning that the *p* value threshold was 0.00093. In the atrophy analysis there was no strong evidence of an effect of polygenic risk of depression on 12‐month regional atrophy (see Supporting Information S1: Tables s5 & s7). This main finding was not changed by controlling for *APOE* genotype (see Supporting Information S1: Table s6).

### 3.5 Meta‐Analysis

The atrophy data was meta‐analysed using a random effects meta‐analysis in R. It should be noted that the parcellation was not identical in ROSMAP and ADNI, meaning that there are likely to be small differences in the areas under investigation. It should also be noted that there were differences in recruitment criteria between the two studies, for example ROSMAP recruited people without known dementia at baseline, whereas ADNI recruited people with normal cognition, MCI and also early dementia As far as possible the same co‐variates were used in both analyses and the PRS‐D was constructed identically and only included SNPs available in both datasets. The confidence intervals were notably much wider in ROSMAP, which is a function of the lower numbers in this dataset. There was no evidence from the meta‐analysis of an effect of PRS‐D on regional brain atrophy (see Figure 3).

FIGURE 3Random effects meta‐analysis of the effect of polygenic risk of depression on regional brain volume change per year in ROSMAP and 12 month regional atrophy in ADNI. Red boxes represent point estimate (odds ratio) and horizontal lines the 95% confidence intervals. Black diamonds are pooled estimates for each risk factor. Estimates above 0 (on the right‐hand side of the *y*‐axis) suggest an increase in brain volume over time, while estimates below 0 (on the left‐hand side of the *y*‐axis) suggests a decrease in brain volume over time. Please note that each brain area was analysed separately for left and right.

**Figure:**
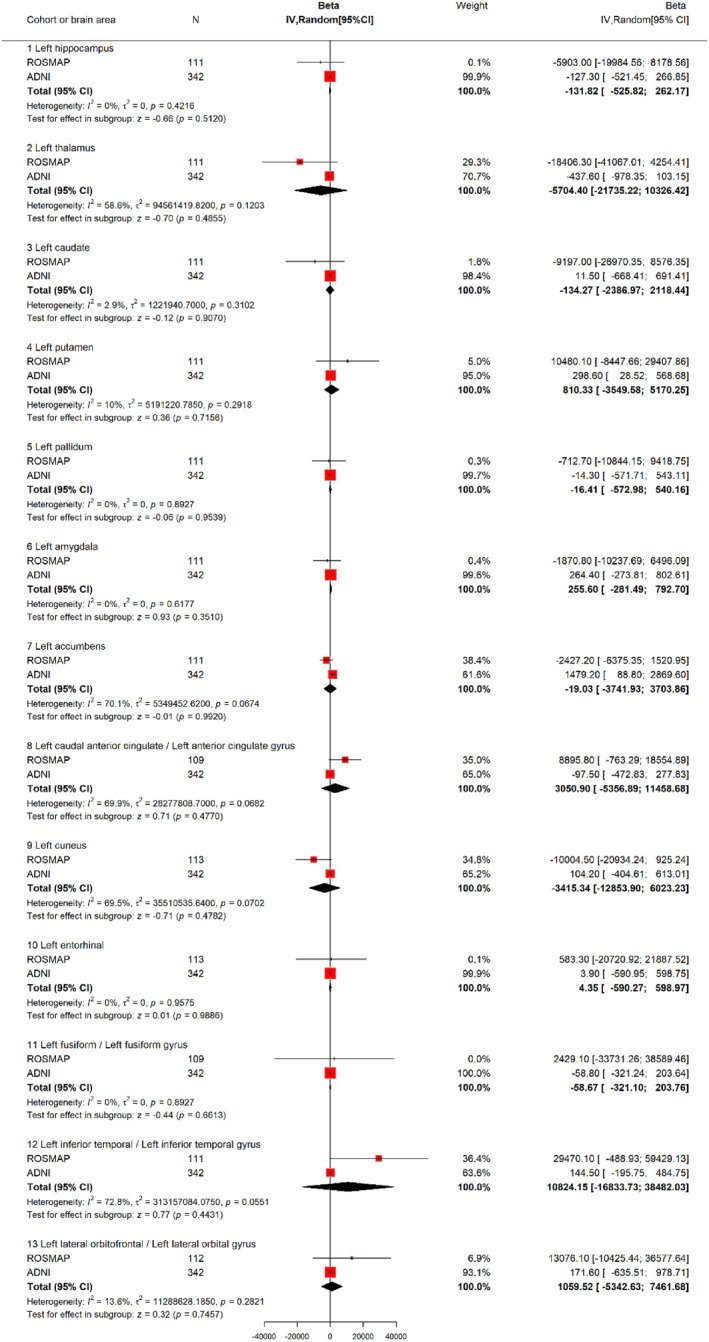

**Figure:**
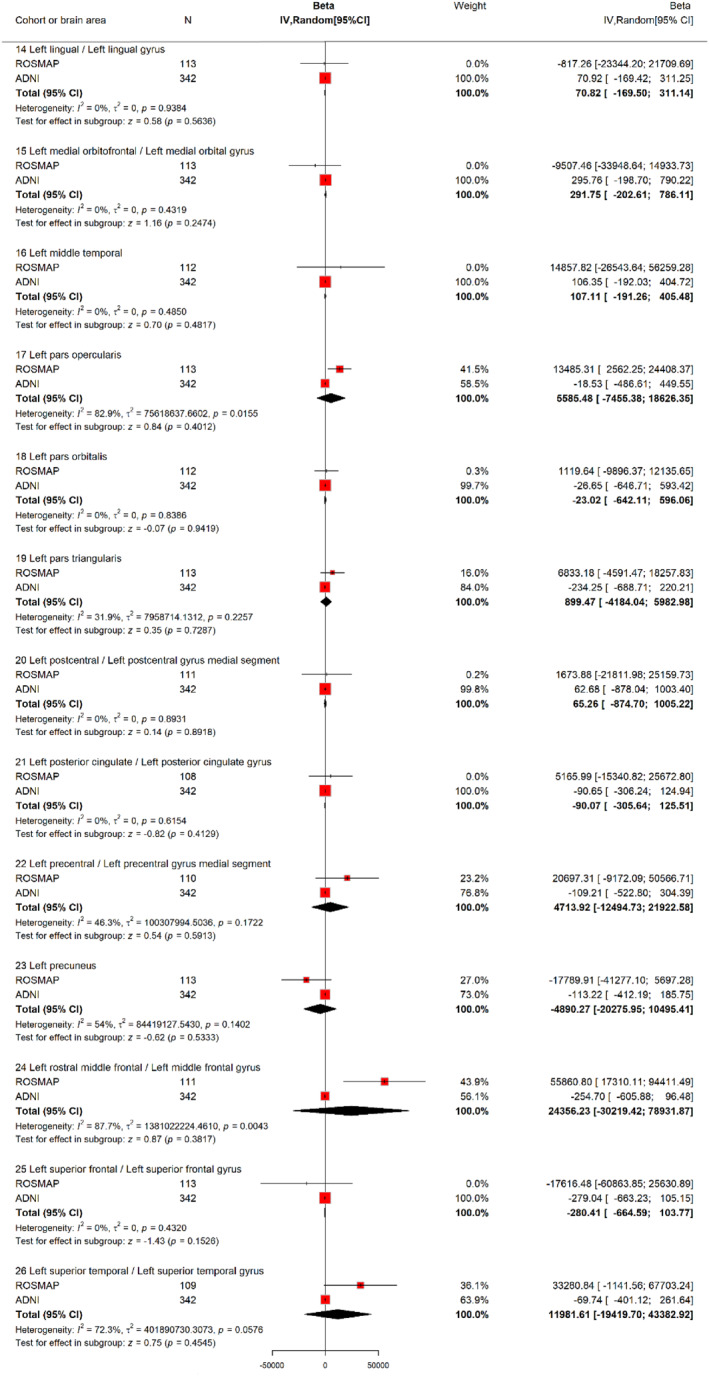

**Figure:**
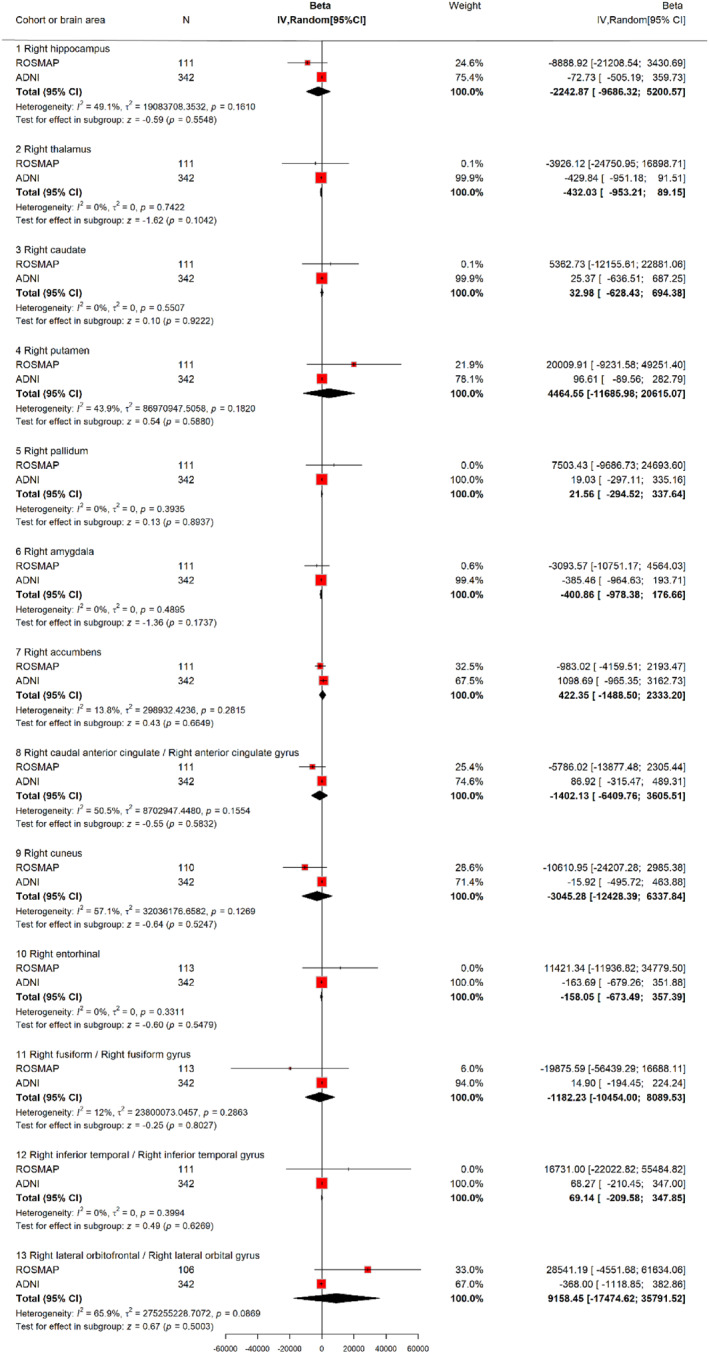

**Figure:**
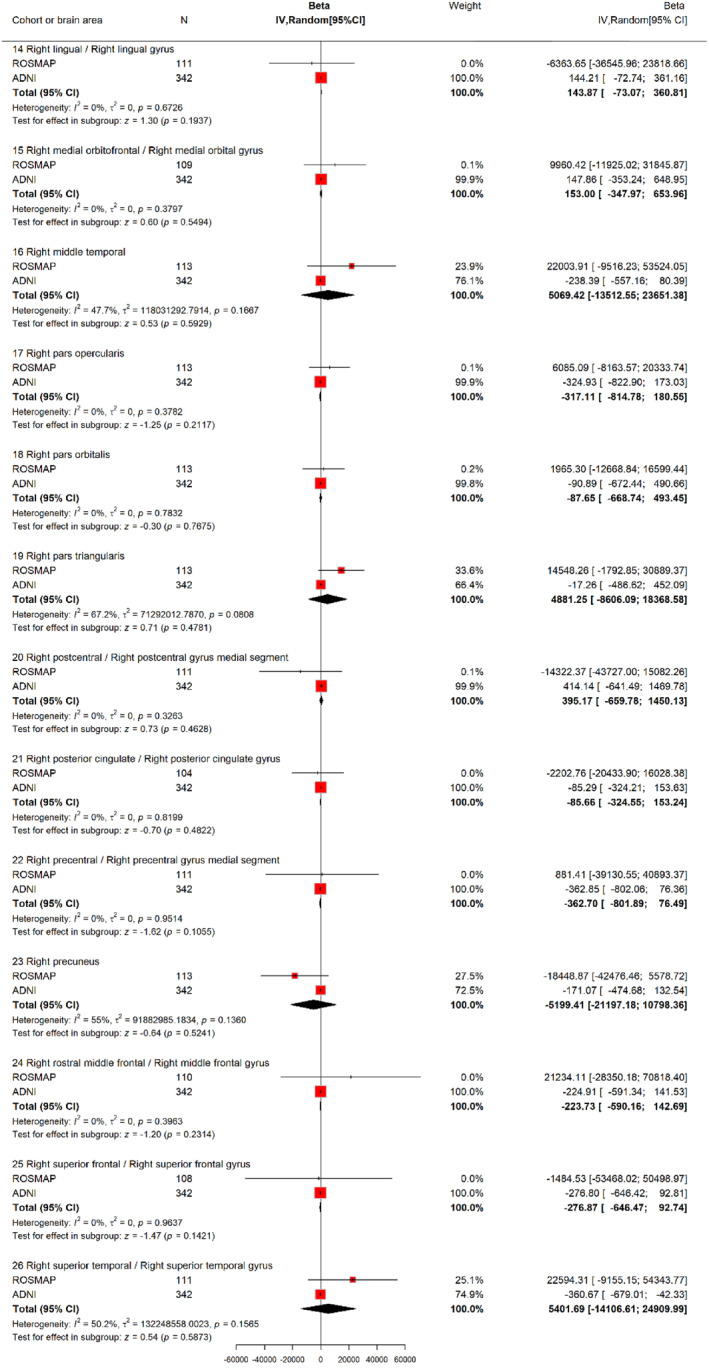

## 4 Discussion

In this study we aimed to use genetic risk of depression to identify the effect of depression on brain volumes and to assess whether higher genetic risk of depression is associated with greater regional brain atrophy over time. We used data from ROSMAP and ADNI and together these studies met our power calculation requirement. We did not find any evidence of an effect of polygenic risk for depression on regional brain volume changes after 12 months, either in ROSMAP or ADNI individually or in the meta‐analysis. There was no evidence of an effect of PRS‐D on regional brain volumes at baseline in either study (ROSMAP *n* = 187, ADNI *n* = 473) and this finding was not affected by excluding individuals with evidence of dementia at baseline in ADNI or in ROSMAP those with AD at post‐mortem examination.

Depression has been identified by the Lancet commission for dementia as a potentially modifiable risk factor for dementia, but the mechanism underlying this is unknown [1]. There is a complex relationship between depression and dementia [1]. Although depression has been identified as a risk factor for dementia some studies suggest that, rather than being a risk factor for AD, later life depression reflects prodromal symptoms of AD [47, 48, 49, 50, 51]. Post‐mortem studies have also attempted to resolve this question. Some studies have found more prominent AD pathology in those with a history of low mood, while others have not. Most of these studies have been relatively small and looked at milder depression [52, 53, 54]. It is likely that AD and depression do not have an overlapping genetic architecture [55], although this has been challenged in a more recent paper [56]. Some of the increase in dementia risk associated with depression may be explained by other modifiable risk factors for dementia. For example, individuals with depression may have disrupted sleep, are more likely to smoke, may be less physically active and are less likely to be motivated to eat a healthy diet [57, 58, 59].

Some, but not all previous neuroimaging studies in this area have reported an effect of depression on brain volumes such as more widespread cortical thinning in those with a history of depression [60, 61]. Hippocampal atrophy in older adults with depression has been shown in some but not all studies [9, 10, 11, 12, 13, 62, 63, 64]. Some studies have also included other brain areas and have had conflicting findings. In a well performed cross sectional study (*n* = 610) Ancelin et al. found that older adults with a history of later life depression have increased gray matter volume in the rostral ACC, lingual and pericalcarine regions [65]. A smaller cross‐sectional MRI study (*n* = 24 early onset depression *n* = 25 later life depression, *n* = 49 controls) found that lifetime depression was associated with thinning of the left fusiform, parahippocampal and inferior temporal regions but that this was not seen when depression was restricted to later life depression only [66]. In a further relatively small cross‐sectional MRI study (*n* = 30 patients and *n* = 22 controls) older adults with depression had decreased gray matter volume in the orbitofrontal cortex [67]. In a small longitudinal study (*n* = 61) of depressed older adults the *APOE* ε4 allele appeared to interact with baseline reductions in left hippocampal volume to affect cognitive decline at 4 year follow‐up [68]. Some of the discrepancies in these findings could be due to the relatively small size of many of these studies, or due to differences in brain regions under investigation.

Taken together, the existing literature suggest that later life depression does have effects on regional brain volume, but in our analyses we did not find an effect of polygenic risk of depression. It is increasingly recognized that affective symptoms including depression can be among the earliest symptoms of neurodegeneration [69], but it can be difficult to tease out new onset symptoms as opposed to those from recurrent depressive illnesses which began earlier in life. Depression, as discussed previously, has been identified as a risk factor for dementia by the Lancet Commission [1], but the mechanism for this risk is unknown. Not all studies have found that depression in the years preceding a dementia diagnosis is due to the dementia itself [70, 71] suggesting a complex relationship between the two conditions which is difficult to dissect. Our findings could equally suggest that while the depression itself may not cause the volumetric changes, there may be something about the depressed brain that ultimately leads to these changes. Such changes could include vascular dysfunction (e.g., the long standing vascular depression hypothesis [72]) or neuroinflammation [73]. It is also possible that volume changes are driven by the length of depressive illness or behavioral changes which occur in depression such as reduced physical activity, social withdrawal and lack of involvement in activities.

Strengths of this study include combining data from two cohorts, being able to examine data from multiple brain regions, including individuals from non‐European genetic backgrounds, the wealth of clinical and demographic data from both cohorts and the high quality genetic data available. We assessed a range of thresholds in the PRS calculation and because we used the vcf files for the clumping and threshold were able to include the level of certainty about each imputed SNP genotype, instead of making hard calls. Weaknesses include the short follow‐up period (12 months), that neither study alone had sufficient individuals to meet our power calculation requirement of *n* = 400 for the atrophy analysis (ADNI *n* = 342), the slight differences in brain parcellation in the two studies and the minor differences in covariates in the two studies. For example, ROSMAP had data on diabetes, but in ADNI this was collapsed into endocrine disease present/absent. ADNI also excluded individuals with current depression, or depression in the last 2 years at the recruitment stage, resulting in low numbers of individuals with depressive symptoms at baseline and potentially decoupling the polygenic risk of depression from the depression phenotype. The small number of studies (only two) in our meta‐analysis limits the conclusions that can be drawn from this study's results and replication will be required. The GWAS that we used to calculate our PRS‐depression included individuals from a wide range of ages, but predominantly younger adults so may be less relevant to later life depression. Although we included individuals from non‐European genetic backgrounds in our analyses (and included the first 4 principal components from the population stratification analysis as a co‐variates) both cohorts have a preponderance of Caucasian participants. More diverse cohorts with suitable data are not currently available. ADNI had a larger number of participants at baseline with dementia or MCI, as a result of its study design but we included cognitive status at baseline as a covariate in the ADNI analyses to attempt to account for this. We have not formally estimated heterogeneity due to the low number of studies meta‐analysed, but it is likely that heterogeneity between studies could have affected our findings. Future studies should include a larger number of participants, and participants from more diverse ethnic backgrounds.

In conclusion we found no strong evidence of an effect of polygenic risk of depression on regional brain volumes either at baseline or after 12 months in older adults. Whilst our work was limited to the effects of genetic risk of depression, it is possible that previously demonstrated effects of depression on regional brain volumes may be due to incipient neurodegeneration, rather than the effects of depression itself. Future studies should include larger numbers of individuals and assess similar brain areas so that the results can be combined with the results of this and other studies in meta‐analyses.

## Funding

Dr Sinclair is funded by a junior fellowship from the Alzheimer's Society (Grant 518) and also by the Elizabeth Blackwell Institute at the University of Bristol and the Wellcome Trust Institutional Strategic Support Fund (204813/Z/16/Z). This project was part funded by the Hannah Steinberg award from the British Association of Psychopharmacology and by a minor travel grant from the Dowager Countess Eleanor Peel Trust. Professor Ballard and Dr Palmer are funded by the NIHR. Sarah Medland is funded by Investigator grants from the Australian National Health and Medical Research Council (APP1172917 and APP 2025674). Data collection and sharing for the Alzheimer's Disease Neuroimaging Initiative (ADNI) is funded by the National Institute on Aging (National Institutes of Health Grant U19AG024904). The grantee organization is the Northern California Institute for Research and Education. In the past, ADNI has also received funding from the National Institute of Biomedical Imaging and Bioengineering, the Canadian Institutes of Health Research, and private sector contributions through the Foundation for the National Institutes of Health (FNIH) including generous contributions from the following: AbbVie, Alzheimer's Association; Alzheimer's Drug Discovery Foundation; Araclon Biotech; BioClinica Inc.; Biogen; BristolMyers Squibb Company; CereSpir Inc.; Cogstate; Eisai Inc.; Elan Pharmaceuticals Inc.; Eli Lilly and Company; EuroImmun; F. Hoffmann‐La Roche Ltd and its affiliated company Genentech Inc.; Fujirebio; GE Healthcare; IXICO Ltd.; Janssen Alzheimer Immunotherapy Research & Development LLC.; Johnson & Johnson Pharmaceutical Research & Development LLC.; Lumosity; Lundbeck; Merck & Co. Inc.; Meso Scale Diagnostics LLC.; NeuroRx Research; Neurotrack Technologies; Novartis Pharmaceuticals Corporation; Pfizer Inc.; Piramal Imaging; Servier; Takeda Pharmaceutical Company; and Transition Therapeutics. The ROSMAP study was funded by the National Institute of Aging (P30AG072975, R01AG017917, P30AG072975, R01AG015819, and P30AG010161). A full list of all grants which have contributed to the ROSMAP data collection can be found at https://www.radc.rush.edu/docs/radcGrants.htm.

## Ethics Statement

Both ROS and MAP were approved by an Institutional Review Board of Rush University Medical Center and all participants signed an Anatomic Gift Act as well as repository and informed consents. ADNI obtained ethical approval from the Institutional Review Boards of all of the participating institutions. Both studies obtained informed consent from participants at the point of study entry, including for secondary use of their data. As this study's use of ADNI and ROSMAP data fell into the category of secondary analysis of anonymised data, under UK law, no separate ethical approval was required for these analyses.

## Conflicts of Interest

In the last 5 years Dr Sinclair has received funding from the Alzheimer's Society (Grant 518), the Elizabeth Blackwell Institute at the University of Bristol and the Wellcome Trust Institutional Strategic Support Fund (204813/Z/16/Z), Alzheimer's Research UK (ARUK‐PPG2022B‐020), the David Telling Charitable Trust, BRACE Alzheimer's Research and the British Neuropathological Society. Professors Bennett and Medland have no relevant conflicts of interest to declare. Dr Palmer has received funding from a British Heart Foundation accelerator award (AA/18/7/34,219) and works in a unit that is supported by the University of Bristol and UK Medical Research Council (MC_UU_00032/5). She has no other relevant conflicts of interest to disclose. During the last 3 years, Clive Ballard has received consulting fees from Acadia pharmaceutical company, AARP, Addex pharmaceutical company, Eli Lily, Enterin pharmaceutical company, GWPharm, H.Lundbeck pharmaceutical company, Novartis pharmaceutical company, Janssen Pharmaceuticals, Johnson and Johnson pharmaceuticals, Novo Nordisk pharmaceutical company, Orion Corp pharmaceutical company, Otsuka America Pharm Inc., Sunovion Pharm. Inc., Suven pharmaceutical company, Roche pharmaceutical company, Biogen pharmaceutical company, Synexus clinical research organization and tauX pharmaceutical company and research funding from synexus clinical research organization, Roche pharmaceutical company, Novo Nordisk pharmaceutical company, Novartis pharmaceutical company, Medical research council (UK), Wellcome trust (UK), National Institute for Health Research (UK), National Institute for Health (US), IMI (Eu), Michael J Fox foundation (US), Alzheimer's Disease Drug Discovery foundation (US), Alzheimer's Society (UK), Parkinson's Society (UK), Alzheimer's Research UK, the Gilling's foundation and BRACE (UK).

## Supporting information


Supporting Information S1

## Data Availability

The ROSMAP GWAS data used in this project is available free of charge from NIAGADS https://dss.niagads.org/cohorts/religious‐orders‐study‐memory‐and‐aging‐project‐rosmap/ to bona fide researchers, subject to approval. The ROSMAP phenotypic data used in this project is available to bona fide researchers who apply to the RARC Research Resource Sharing Hub (https://www.radc.rush.edu/). The ADNI genetic and phenotypic data used in this study was obtained from ADNI (www.loni.ucla.edu/ADNI) and is available free of charge to bona fide researchers who submit a research proposal.
