# Supplementary material for: The Relationship Between Polygenic Risk of Depression and Regional Brain Atrophy in Older Adults
Source: Int J Geriatr Psychiatry. 2026 Aug 16;41(8):e70245. doi: 10.1002/gps.70245 (PMC13477798; doi:10.1002/gps.70245)
Supplement: Supplementary file 1 [file GPS-41-e70245-s001.docx]

[Table s1: The effect of PRS-D on regional brain volumes (mm3) at baseline in the ROSMAP cohort.	2](#_Toc233789207)

[Table s2: The effect of PRS-D on regional brain volumes (mm3) at baseline in the ROSMAP cohort, excluding those with post-mortem evidence of AD.	4](#_Toc233789208)

[Table s3: The effect of PRS-D on regional brain atrophy over time in the ROSMAP cohort.	6](#_Toc233789209)

[Table s4: The effect of PRS-D on regional brain volumes (mm3) at baseline in the ADNI cohort.	8](#_Toc233789210)

[Table s5: The effect of PRS-D on regional brain atrophy at 12 months in the ADNI cohort.	10](#_Toc233789211)

[Table s6: Secondary analysis of the effect of PRS-D on regional brain atrophy at 12 months in the ADNI cohort, controlling for APOE genotype.	12](#_Toc233789212)

[Table s7: Secondary analysis of the effect of PRS-D on regional brain volumes (mm3) at baseline in individuals without dementia at baseline in the ADNI cohort.	14](#_Toc233789213)

Table s1: The effect of PRS-D on regional brain volumes (mm3) at baseline in the ROSMAP cohort. In this analysis all individuals in the ROSMAP cohort with a PRS-D and MRI data were included, regardless of cognitive status. Note all analyses adjusted for sex, first 4 principal components from the population stratification analysis, baseline age, marital status, smoking status, heaviest lifetime drinking, years of education and lifetime presence of hypertension or diabetes.

| **Brain Area**  **(total volume in mm^3^)** | **Beta co-efficient** | **95%CI** | **P value** | **R^2^** |
| --- | --- | --- | --- | --- |
| Right hippocampus | -74191.9 | -156166.8 – 7783.1 | 0.076 | 0.374 |
| Left hippocampus | -37414.1 | -116327.7 – 41499.5 | 0.351 | 0.273 |
| Right thalamus | -8148.3 | -112555.6 – 96259.0 | 0.878 | 0.256 |
| Left thalamus | 37730.9 | -68406.3 – 143868 | 0.484 | 0.246 |
| Right caudate | 107174.4 | -1525.5 – 215874.3 | 0.053 | 0.330 |
| Left caudate | 116084.4 | -1936.1 – 234104.9 | 0.054 | 0.298 |
| Right putamen | 21156.8 | -88772.0 - 131085.6 | 0.704 | 0.112 |
| Left putamen | 29057.3 | -79594.7 – 137709.3 | 0.598 | 0.147 |
| Right pallidum | 39140.2 | -3223.6 – 81503.9 | 0.070 | 0.215 |
| Left pallidum | 22894.9 | -18716.3 – 64506.0 | 0.279 | 0.199 |
| Right amygdala | -12866.0 | -55513.8 – 29781.8 | 0.552 | 0.199 |
| Left amygdala | -18870.3 | -58156.8 – 20416.3 | 0.344 | 0.334 |
| Right accumbens | -8306.6 | -22914.7 – 6301.6 | 0.263 | 0.181 |
| Left accumbens | -4025.8 | -17620.5 – 9568.9 | 0.559 | 0.240 |
| Right ventral dc | -24644.2 | -81751.3 – 32462.8 | 0.395 | 0.283 |
| Left ventral dc | -9302.6 | -71644.8 – 53039.7 | 0.769 | 0.314 |
| Right caudal anterior cingulate | -48057.8 | -120220 – 24104.5 | 0.190 | 0.163 |
| Left caudal anterior cingulate | -49846.6 | -119999.9 – 20306.8 | 0.162 | 0.165 |
| Right caudal middle frontal | 13014.7 | -141634.8 – 167664.2 | 0.868 | 0.254 |
| Left caudal middle frontal | 10474.0 | -129912 – 150859.9 | 0.883 | 0.272 |
| Right cuneus | 66508.5 | -13409.4 – 146426.5 | 0.102 | 0.220 |
| Left cuneus | 77459.0 | 1964.8 – 152953.2 | 0.044 | 0.247 |
| Right entorhinal | 7819.5 | -66334.0 – 81973.1 | 0.835 | 0.131 |
| Left entorhinal | 29851.8 | -38593.3 – 98296.9 | 0.390 | 0.195 |
| Right fusiform | 76067.7 | -122917.5 – 275052.9 | 0.451 | 0.237 |
| Left fusiform | 13123.0 | -166191 – 192436.0 | 0.885 | 0.366 |
| Right inferior parietal | 132365.3 | -153611.5 – 418342.1 | 0.362 | 0.264 |
| Left inferior parietal | 24318.6 | -226653.8 – 275290.9 | 0.849 | 0.229 |
| Right inferior temporal | 54180.4 | -179003.3 – 287364 | 0.647 | 0.273 |
| Left inferior temporal | -72263.8 | -315295.8 – 170768.3 | 0.558 | 0.298 |
| Right isthmus cingulate | 7320.4 | -52557.7 – 67198.6 | 0.810 | 0.201 |
| Left isthmus cingulate | 4913.7 | -58195.6 – 68023.1 | 0.878 | 0.351 |
| Right lateral orbital frontal | -19692.7 | -143555.5 – 104170 | 0.754 | 0.279 |
| Left lateral orbital frontal | -35956.1 | -159559.4 – 87647.2 | 0.566 | 0.262 |
| Right lingual | 5225.9 | -157265 – 167717 | 0.949 | 0.270 |
| Left lingual | 104205.9 | -54487.7 – 262899.5 | 0.197 | 0.260 |
| Right medial orbitofrontal | -37586.7 | -130829.7 – 55656.4 | 0.427 | 0.212 |
| Left medial orbitofrontal | 57251.9 | - 33643.9 – 148147.7 | 0.215 | 0.262 |
| Right middle temporal | -61100.4 | -300161.2 – 177960.4 | 0.614 | 0.283 |
| Left middle temporal | -8446.3 | -235846.1 – 218953.4 | 0.942 | 0.257 |
| Right parahippocampal | -25559.1 | -73142.8 – 22024.6 | 0.290 | 0.167 |
| Left parahippocampal | -8277.1 | -64470.6 – 47916.4 | 0.772 | 0.152 |
| Right paracentral | 3433.7 | -82255.7 – 89123.0 | 0.937 | 0.203 |
| Left paracentral | 38895.9 | -34787.3 – 112579.1 | 0.299 | 0.200 |
| Right pars opercularis | -20650 | -104555.8 – 63255.9 | 0.628 | 0.244 |
| Left pars opercularis | -78595.4 | -181921.7 – 24730.9 | 0.135 | 0.234 |
| Right pars orbitalis | -31816.5 | -86753.3 – 23120.3 | 0.254 | 0.199 |
| Left pars orbitalis | 12115.4 | -29260.2 – 53490.9 | 0.564 | 0.235 |
| Right pars triangularis | -28148.1 | -130290.4 – 73994.3 | 0.587 | 0.134 |
| Left pars triangularis | -35825.7 | - 113905.4 – 42254.0 | 0.366 | 0.233 |
| Right pericalcarine | 47985.6 | -27385.5 – 123356.8 | 0.210 | 0.188 |
| Left pericalcarine | 60989.1 | -7670.5 – 129648.7 | 0.081 | 0.194 |
| Right postcentral | 75960.9 | -113746.3 – 265668.2 | 0.430 | 0.281 |
| Left postcentral | 41940.3 | -132477.5 – 216358.1 | 0.636 | 0.337 |
| Right posterior cingulate | 2270.0 | -83735.3 – 88275.3 | 0.958 | 0.253 |
| Left posterior cingulate | -25074.3 | -107194.1 – 57045.5 | 0.547 | 0.185 |
| Right precentral | -8330.6 | -245341.3 – 228680 | 0.945 | 0.295 |
| Left precentral | -42575.4 | -283113.4 – 19762.6 | 0.727 | 0.289 |
| Right precuneus | -11604.8 | - 185361.4 – 162151.9 | 0.895 | 0.391 |
| Left precuneus | -44775.1 | -218847.5 – 129297.4 | 0.612 | 0.384 |
| Right rostral anterior cingulate | -30614.0 | -100234.1 – 39006.1 | 0.387 | 0.170 |
| Left rostral anterior cingulate | -13708.36 | -99551.8 – 72135.1 | 0.753 | 0.248 |
| Right rostral middle frontal | -20304.2 | -301874.4 – 261266 | 0.887 | 0.371 |
| Left rostral middle frontal | 4459.8 | -285946.4 – 294866 | 0.976 | 0.313 |
| Right superior frontal | 153284.5 | -213946.7 – 520515.6 | 0.411 | 0.334 |
| Left superior frontal | 133741.5 | -251358.2 – 518841.2 | 0.494 | 0.300 |
| Right superior parietal | -43357.4 | -282772.3 – 196057.5 | 0.721 | 0.366 |
| Left superior parietal | -34724.1 | -273471.7 – 204023.5 | 0.774 | 0.368 |
| Right superior temporal | -9031.8 | -242060 – 223996.3 | 0.939 | 0.241 |
| Left superior temporal | 49680.4 | -185481 – 284842 | 0.677 | 0.243 |
| Right insula | -5553.1 | -145933.9 – 134827.7 | 0.938 | 0.271 |
| Left insula | -15792.6 | -158164.1 – 126578.9 | 0.827 | 0.292 |
| Right transverse temporal | 1914.8 | -24615.7 – 28445.2 | 0.887 | 0.249 |
| Left transverse temporal | -15492.8 | -52098.4 – 21112.9 | 0.405 | 0.206 |

Table s2: The effect of PRS-D on regional brain volumes (mm3) at baseline in the ROSMAP cohort, excluding those with post-mortem evidence of AD. Note all analyses adjusted for sex, first 4 principal components from the population stratification analysis, baseline age, marital status, smoking status, heaviest lifetime drinking, years of education and lifetime presence of hypertension or diabetes.

| **Brain Area**  **(total volume in mm^3^)** | **Beta co-efficient** | **95%CI** | **P value** | **R^2^** |
| --- | --- | --- | --- | --- |
| Right hippocampus | -79898.8 | -179364.3 – 19566.7 | 0.114 | 0.316 |
| Left hippocampus | -41526.0 | -131822.9 – 48763.0 | 0.364 | 0.396 |
| Right thalamus | -20033.9 | -143980.4 - 103912.6 | 0.749 | 0.338 |
| Left thalamus | 14697.4 | -112304.1 - 141699 | 0.819 | 0.306 |
| Right caudate | 78231.2 | -41380.0 – 197842.4 | 0.198 | 0.152 |
| Left caudate | 102152.8 | -28079.8 – 232385.4 | 0.123 | 0.179 |
| Right putamen | -16835.7 | -135950.2 -102278.9 | 0.780 | 0.187 |
| Left putamen | 8540.2 | -125324 - 142404.3 | 0.900 | 0.172 |
| Right pallidum | 45154.1 | -3631.2 – 93939.3 | 0.069 | 0.287 |
| Left pallidum | 31495.0 | -17459.4 - 80449.3 | 0.205 | 0.285 |
| Right amygdala | -23220.5 | -71788.6 - 25347.7 | 0.345 | 0.273 |
| Left amygdala | -24983.0 | -72746.9 - 22780.9 | 0.302 | 0.329 |
| Right accumbens | -3372.0 | -21376.4 - 14632.4 | 0.711 | 0.198 |
| Left accumbens | -4110.0 | -20391.4 -12171.5 | 0.618 | 0.291 |
| Right ventral dc | -16837.6 | -82660.4 - 48985.3 | 0.613 | 0.389 |
| Left ventral dc | -9303.5 | -82424.9 - 63817.8 | 0.801 | 0.391 |
| Right caudal anterior cingulate | -29078.2 | -112751.6 - 54595.1 | 0.492 | 0.228 |
| Left caudal anterior cingulate | -51337.5 | -138911.6 - 36236.5 | 0.248 | 0.152 |
| Right caudal middle frontal | 7989.0 | -175878.8 - 191856.8 | 0.932 | 0.342 |
| Left caudal middle frontal | 22285.1 | -139807.4 - 184377.6 | 0.786 | 0.363 |
| Right cuneus | 70761.5 | -28688.8 - 170211.9 | 0.161 | 0.240 |
| Left cuneus | 85748.7 | -8877.1 - 180374.4 | 0.075 | 0.250 |
| Right entorhinal | 8926.7 | -80833.5 – 98686.9 | 0.844 | 0.153 |
| Left entorhinal | 32021.6 | -46156.4 – 110199.7 | 0.419 | 0.277 |
| Right fusiform | 23321.7 | -206153.6 - 252796.9 | 0.841 | 0.325 |
| Left fusiform | 19832.5 | -188678.7 - 228343.6 | 0.851 | 0.416 |
| Right inferior parietal | 175570.7 | -163962 .0 - 515103.3 | 0.308 | 0.340 |
| Left inferior parietal | 17048.9 | -284569.8 - 318667.7 | 0.911 | 0.309 |
| Right inferior temporal | 58791.2 | -222572.6 – 340155.0 | 0.690 | 0.303 |
| Left inferior temporal | -22477.6 | -308757.1 - 263801.9 | 0.877 | 0.348 |
| Right isthmus cingulate | 31143.8 | -41708.2 - 103995.8 | 0.399 | 0.229 |
| Left isthmus cingulate | -868.7 | -75579.3 - 73841.9 | 0.982 | 0.399 |
| Right lateral orbitofrontal | -18896.9 | -169520 – 131726.2 | 0.804 | 0.344 |
| Left lateral orbitofrontal | -26191.8 | -176905.2 – 124521.7 | 0.731 | 0.325 |
| Right lingual | 7937 | -185892 .0 –201766.0 | 0.935 | 0.341 |
| Left lingual | 100672.9 | -90356.3 - 291702.1 | 0.299 | 0.327 |
| Right medial orbitofrontal | -41301.0 | -142851.8 – 60249.9 | 0.422 | 0.359 |
| Left medial orbitofrontal | 26629.2 | -81518.0 – 134776.4 | 0.627 | 0.319 |
| Right middle temporal | -76034.1 | -350036.0 - 197967.8 | 0.583 | 0.360 |
| Left middle temporal | -64781.3 | -329956.9 - 200394.3 | 0.629 | 0.304 |
| Right parahippocampal | -22408.0 | -76794.6 - 31978.6 | 0.416 | 0.219 |
| Left parahippocampal | -11735.8 | -76508.3 - 53036.8 | 0.720 | 0.333 |
| Right paracentral | -45220.3 | -147624.7 - 57184.0 | 0.383 | 0.265 |
| Left paracentral | 45943.5 | -47639.7 - 139526.7 | 0.333 | 0.229 |
| Right pars opercularis | -28403.9 | -123842.6 - 67034.8 | 0.557 | 0.352 |
| Left pars opercularis | -104667.2 | -231867.6 - 22533.1 | 0.106 | 0.283 |
| Right pars orbitalis | -36630.0 | -98360.4 - 25100.5 | 0.242 | 0.263 |
| Left pars orbitalis | 19273.2 | -31168.9 - 69715.3 | 0.451 | 0.262 |
| Right pars triangularis | -44314.3 | -149426.2 - 60797.7 | 0.405 | 0.201 |
| Left pars triangularis | -38323.4 | -128716.8 – 52070.0 | 0.403 | 0.277 |
| Right pericalcarine | 55751.9 | -36413.0 - 147916.7 | 0.233 | 0.234 |
| Left pericalcarine | 59688.0 | -23761.9 - 143137.9 | 0.159 | 0.225 |
| Right postcentral | 54839.3 | -171994.0 - 281672.6 | 0.633 | 0.313 |
| Left postcentral | 6902.6 | -207977.4 - 221782.5 | 0.949 | 0.364 |
| Right posterior cingulate | 30002.3 | -62910.0 - 122914.7 | 0.523 | 0.320 |
| Left posterior cingulate | -13705.1 | -104724.4 -77314.2 | 0.766 | 0.269 |
| Right precentral | 2648.6 | -283653.9 - 288951.1 | 0.985 | 0.352 |
| Left precentral | -66407.2 | -360666.3 – 227852.0 | 0.656 | 0.340 |
| Right precuneus | -56537.6 | -267641.6 - 154566.3 | 0.597 | 0.481 |
| Left precuneus | -56441.2 | -271824.7 -158942.4 | 0.605 | 0.327 |
| Right rostral anterior cingulate | 3853.7 | -76403.4 - 84110.8 | 0.924 | 0.254 |
| Left rostral anterior cingulate | -4623.6 | -105834.8 - 96587.7 | 0.928 | 0.325 |
| Right rostral middle frontal | 93460.1 | -228878.3 - 415798.5 | 0.567 | 0.471 |
| Left rostral middle frontal | 19099.3 | -325218.4 – 363417.0 | 0.913 | 0.368 |
| Right superior frontal | 103165.4 | -327374.5 - 533705.3 | 0.636 | 0.404 |
| Left superior frontal | 133871.7 | -341806.6 – 609550.0 | 0.578 | 0.368 |
| Right superior parietal | -54237.3 | -348000.4 - 239525.7 | 0.715 | 0.328 |
| Left superior parietal | -38894.6 | -322154.3 - 244365.1 | 0.786 | 0.354 |
| Right superior temporal | -11990.9 | -288221.0 - 264239.2 | 0.932 | 0.320 |
| Left superior temporal | 81982.6 | -197991.4 - 361956.7 | 0.563 | 0.318 |
| Right insula | 13355.5 | -143463.2 - 170174.2 | 0.866 | 0.404 |
| Left insula | -54885.7 | -215545.6 - 105774.3 | 0.500 | 0.407 |
| Right transverse temporal | 8740.3 | -22637.5 - 40118.0 | 0.582 | 0.351 |
| Left transverse temporal | -18182.0 | -62370.8 - 26006.7 | 0.417 | 0.272 |

Table s3: The effect of PRS-D on regional brain atrophy over time in the ROSMAP cohort. In this analysis all individuals in the ROSMAP cohort with a PRS-D and MRI data from 2 scans were included, regardless of cognitive status (n=113). Note all analyses adjusted for sex, first 4 principal components from the population stratification analysis, baseline age, marital status, smoking status, heaviest lifetime drinking, years of education and lifetime presence of hypertension or diabetes. Atrophy was calculated as the difference between the volume at scan 2 and scan 1, divided by the time difference in years.

| **Brain Area**  **(atrophy per year mm^3^)** | **Beta co-efficient** | **Lower 95% CI** | **Upper 95%CI** | **P value** | **R^2^** |
| --- | --- | --- | --- | --- | --- |
| Right hippocampus | -8888.9 | -21382.3 | 3604.4 | 0.161 | 0.252 |
| Left hippocampus | -5903.0 | -20183.1 | 8377.1 | 0.414 | 0.156 |
| Right thalamus | -3926.1 | -25044.7 | 17192.4 | 0.713 | 0.172 |
| Left thalamus | -18406.3 | -41386.6 | 4574.1 | 0.115 | 0.202 |
| Right caudate | 5362.7 | -12402.7 | 23128.2 | 0.55 | 0.311 |
| Left caudate | -9197.0 | -27221.1 | 8827.1 | 0.313 | 0.209 |
| Right putamen | 20009.9 | -9644.0 | 49663.8 | 0.183 | 0.163 |
| Left putamen | 10480.1 | -8714.6 | 29674.8 | 0.281 | 0.218 |
| Right pallidum | 7503.4 | -9929.2 | 24936.1 | 0.395 | 0.104 |
| Left pallidum | -712.7 | -10987.0 | 9561.7 | 0.891 | 0.149 |
| Right amygdala | -3093.6 | -10859.2 | 4672.0 | 0.431 | 0.133 |
| Left amygdala | -1870.8 | -10355.8 | 6614.1 | 0.662 | 0.202 |
| Right accumbens | -983.0 | -4204.3 | 2238.3 | 0.546 | 0.117 |
| Left accumbens | -2427.2 | -6431.1 | 1576.7 | 0.232 | 0.186 |
| Right caudal anterior cingulate | -5786.0 | -13991.6 | 2419.6 | 0.165 | 0.244 |
| Left caudal anterior cingulate | 8895.8 | -902.8 | 18694.4 | 0.075 | 0.209 |
| Right cuneus | -10611.0 | -24401.3 | 3179.4 | 0.13 | 0.183 |
| Left cuneus | -10004.5 | -21084.9 | 1075.9 | 0.076 | 0.218 |
| Right entorhinal | 11421.3 | -12258.8 | 35101.5 | 0.34 | 0.285 |
| Left entorhinal | 583.3 | -21014.5 | 22181.2 | 0.957 | 0.179 |
| Right fusiform | -19875.6 | -56943.3 | 17192.1 | 0.29 | 0.148 |
| Left fusiform | 2429.1 | -34253.4 | 39111.7 | 0.896 | 0.082 |
| Right inferior temporal | 16731.0 | -22569.4 | 56031.4 | 0.4 | 0.274 |
| Left inferior temporal | 29470.1 | -911.4 | 59851.6 | 0.057 | 0.286 |
| Right lateral orbitofrontal | 28541.2 | -5047.3 | 62129.7 | 0.095 | 0.174 |
| Left lateral orbitofrontal | 13076.1 | -10753.1 | 36905.2 | 0.278 | 0.094 |
| Right lingual | -6363.6 | -36971.7 | 24244.4 | 0.68 | 0.115 |
| Left lingual | -817.3 | -23654.7 | 22020.2 | 0.943 | 0.202 |
| Right medial orbitofrontal | 9960.4 | -12241.1 | 32161.9 | 0.375 | 0.155 |
| Left medial orbitofrontal | -9507.5 | -34285.5 | 15270.6 | 0.448 | 0.21 |
| Right middle temporal | 22003.9 | -9950.7 | 53958.5 | 0.175 | 0.271 |
| Left middle temporal | 14857.8 | -27120.9 | 56836.5 | 0.484 | 0.164 |
| Right pars opercularis | 6085.1 | -8360.0 | 20530.1 | 0.405 | 0.205 |
| Left pars opercularis | 13485.3 | 2411.7 | 24558.9 | 0.018 | 0.256 |
| Right pars orbitalis | 1965.3 | -12870.6 | 16801.2 | 0.793 | 0.161 |
| Left pars orbitalis | 1119.6 | -10050.0 | 12289.2 | 0.843 | 0.253 |
| Right pars triangularis | 14548.3 | -2018.1 | 31114.6 | 0.084 | 0.207 |
| Left pars triangularis | 6833.2 | -4748.9 | 18415.3 | 0.244 | 0.129 |
| Right postcentral | -14322.4 | -44141.7 | 15497.0 | 0.342 | 0.28 |
| Left postcentral | 1673.9 | -22143.2 | 25491.0 | 0.889 | 0.183 |
| Right posterior cingulate | -2202.8 | -20713.9 | 16308.4 | 0.813 | 0.224 |
| Left posterior cingulate | 5166.0 | -15640.5 | 25972.5 | 0.623 | 0.092 |
| Right precentral | 881.4 | -39694.9 | 41457.7 | 0.966 | 0.242 |
| Left precentral | 20697.3 | -9598.3 | 50993.0 | 0.178 | 0.214 |
| Right precuneus | -18448.9 | -42807.6 | 5909.9 | 0.136 | 0.19 |
| Left precuneus | -17789.9 | -41600.8 | 6021.0 | 0.141 | 0.193 |
| Right rostral middle frontal | 21234.1 | -29057.8 | 71526.0 | 0.404 | 0.19 |
| Left rostral middle frontal | 55860.8 | 16766.4 | 94955.2 | 0.006 | 0.204 |
| Right superior frontal | -1484.5 | -54227.8 | 51258.7 | 0.955 | 0.118 |
| Left superior frontal | -17616.5 | -61460.0 | 26227.0 | 0.427 | 0.19 |
| Right superior temporal | 22594.3 | -9603.0 | 54791.6 | 0.167 | 0.175 |
| Left superior temporal | 33280.8 | -1638.6 | 68200.3 | 0.061 | 0.271 |
| Right insula | 21736.2 | -9613.5 | 53086.0 | 0.172 | 0.222 |
| Left insula | 34363.4 | 4122.1 | 64804.7 | 0.026 | 0.207 |

Table s4: The effect of PRS-D on regional brain volumes (mm3) at baseline in the ADNI cohort. In this analysis all individuals in ADNI with a PRS-D and MRI data were included, regardless of cognitive status. Note all analyses adjusted for sex, first 4 principal components from the population stratification analysis, baseline age, marital status, smoking status, lifetime history of alcohol abuse, years of education, presence of cardiovascular disease and presence of endocrine disease, n=473.

| **Brain Area**  **(total volume in mm^3^)** | **Beta co-efficient** | **95%CI** | **P value** | **R^2^** |
| --- | --- | --- | --- | --- |
| Right hippocampus | -72.7 | -506.9 - 361.4 | 0.742 | 0.237 |
| Left hippocampus | -40023.7 | -93057.3 - 13009.9 | 0.139 | 0.209 |
| Right thalamus | -7464.1 | -69088.7 - 54160.4 | 0.812 | 0.287 |
| Left thalamus | 2609.7 | -61707.7 - 66927.1 | 0.936 | 0.297 |
| Right caudate | -12889.1 | -64347.9 - 38569.7 | 0.623 | 0.116 |
| Left caudate | -1273.7 | -46545.3 - 43998.0 | 0.956 | 0.112 |
| Right putamen | -13368.4 | -62267.7 - 35531.0 | 0.591 | 0.180 |
| Left putamen | -13545.9 | -62727.6 - 35635.7 | 0.589 | 0.173 |
| Right pallidum | 264.1 | -15340.2 - 15868.4 | 0.973 | 0.222 |
| Left pallidum | -1638.6 | -17763.7 - 14486.4 | 0.842 | 0.204 |
| Right amygdala | -4040.0 | -20858.9 - 12778.9 | 0.637 | 0.193 |
| Left amygdala | -9088.4 | -25423.9 - 7247.0 | 0.275 | 0.224 |
| Right accumbens | -3023.4 | -9880.1 - 3833.2 | 0.387 | 0.135 |
| Left accumbens | -1380.0 | -9304.7 - 6544.6 | 0.732 | 0.121 |
| Right ventral dc | -16555.4 | -58009.5 - 24898.7 | 0.433 | 0.344 |
| Left ventral dc | -17075.4 | -59981.2 - 25830.5 | 0.435 | 0.345 |
| Right anterior cingulate gyrus | -29021.9 | -98835.7 -40791.9 | 0.414 | 0.138 |
| Left anterior cingulate gyrus | -24547.6 | -103826.9 - 54731.7 | 0.543 | 0.154 |
| Right middle frontal gyrus | -35930.9 | -266662.5 - 194800.7 | 0.760 | 0.192 |
| Left middle frontal gyrus | -40673.1 | -273837.5 - 192491.3 | 0.732 | 0.180 |
| Right cuneus | -52474.0 | -132953.1 - 28005.2 | 0.201 | 0.219 |
| Left cuneus | -84484.8 | -164343.9 - -4625.7 | 0.038 | 0.249 |
| Right entorhinal | -16063.3 | -56660.7 - 24534.2 | 0.437 | 0.288 |
| Left entorhinal | -26623.2 | -65920.7 - 12674.4 | 0.184 | 0.252 |
| Right fusiform gyrus | -61733.0 | -176030.9 - 52564.9 | 0.289 | 0.313 |
| Left fusiform gyrus | -37329.1 | -143729.6 - 69071.3 | 0.491 | 0.269 |
| Right inferior temporal gyrus | -137694.8 | -299032.5 - 23642.9 | 0.094 | 0.270 |
| Left inferior temporal gyrus | -64233.6 | -224754.3 - 96287.2 | 0.432 | 0.249 |
| Right lateral orbital gyrus | 2491.9 | -39236.5 - 44220.4 | 0.907 | 0.136 |
| Left lateral orbital gyrus | 8462.9 | -34711.8 - 51637.6 | 0.700 | 0.074 |
| Right lingual gyrus | -46430.0 | -161259.1 - 68399.0 | 0.427 | 0.238 |
| Left lingual gyrus | 2491.9 | -39236.5 - 44220.4 | 0.907 | 0.136 |
| Right medial orbitofrontal | -946.4 | -52291.9 - 50399.1 | 0.971 | 0.271 |
| Left medial orbitofrontal | -16603.2 | -80286.0 - 47079.6 | 0.609 | 0.177 |
| Right middle temporal | -92901.2 | -300119.9 - 114317.6 | 0.379 | 0.174 |
| Left middle temporal | -34945.7 | -242833.1 - 172941.7 | 0.741 | 0.174 |
| Right parahippocampal gyrus | -19733.9 | -62042.3 - 22574.4 | 0.360 | 0.286 |
| Left parahippocampal gyrus | -24876 | -68920.6 -19168.6 | 0.268 | 0.286 |
| Right pars opercularis | 38207.9 | -24488.8 - 100904.6 | 0.232 | 0.126 |
| Left pars opercularis | 62658.9 | 1538.8 - 123779.0 | 0.045 | 0.147 |
| Right pars orbitalis | 2762.5 | -35911.0 - 41436.0 | 0.888 | 0.121 |
| Left pars orbitalis | 10714.3 | -26259.4 - 47687.9 | 0.569 | 0.146 |
| Right pars triangularis | 53426.4 | -2114.0 - 108966.9 | 0.059 | 0.121 |
| Left pars triangularis | 14212.2 | -41675.2 - 70099.6 | 0.617 | 0.119 |
| Right calcarine cortex | -14697.4 | -70903.2 - 41508.3 | 0.608 | 0.156 |
| Left calcarine cortex | -43129.7 | -104471.6 - 18212.3 | 0.168 | 0.164 |
| Right postcentral gyrus | -70697.4 | -200524.8 - 59130.1 | 0.285 | 0.117 |
| Left postcentral gyrus | -58454.1 | -191662.9 - 74754.8 | 0.389 | 0.168 |
| Right posterior cingulate gyrus | -8452.6 | -66680.6 - 49775.4 | 0.776 | 0.202 |
| Left posterior cingulate gyrus | 670.1 | -57162.8 - 58503.1 | 0.982 | 0.227 |
| Right precentral | -192052.9 | -350015.5 - -34090.2 | 0.017 | 0.242 |
| Left precentral | -71054.0 | -229701.0 - 87593.2 | 0.379 | 0.218 |
| Right precuneus | -17231.9 | -182483.2 - 148019.5 | 0.838 | 0.214 |
| Left precuneus | -7699.6 | -172390.7 - 156991.5 | 0.927 | 0.224 |
| Right superior frontal gyrus | -29126.9 | -187968.0 - 129714.2 | 0.719 | 0.208 |
| Left superior frontal gyrus | -1709.5 | -160461.1 - 157042.2 | 0.983 | 0.218 |
| Right superior parietal | -11032.6 | -146918.9 - 124853.6 | 0.873 | 0.162 |
| Left superior parietal | 32799.1 | -114609.8 - 180208.0 | 0.662 | 0.155 |
| Right superior temporal gyrus | -24182.55 | -129670.7 - 81305.6 | 0.653 | 0.140 |
| Left superior temporal gyrus | -37515.2 | -147275.0 - 72244.7 | 0.502 | 0.154 |
| Right anterior insula | -31608.3 | -88911.9 - 25695.3 | 0.279 | 0.221 |
| Left anterior insula | -23504.7 | -80486.8 - 33477.4 | 0.418 | 0.213 |
| Right posterior insula | 3113.7 | -31255.6 - 37483.0 | 0.859 | 0.211 |
| Left posterior insula | 9257.1 | -24076.3 - 42590.5 | 0.585 | 0.198 |
| Right transverse temporal | -23069.4 | -55684.6 - 9545.9 | 0.165 | 0.192 |
| Left transverse temporal | 17578.7 | -20967.4 - 56124.8 | 0.371 | 0.131 |

Table s5: The effect of PRS-D on regional brain atrophy at 12 months in the ADNI cohort. In this analysis all individuals in ADNI with a PRS-D and MRI data were included, regardless of cognitive status. Note all analyses adjusted for sex, first 4 principal components from the population stratification analysis, baseline age, marital status, smoking status, lifetime history of alcohol abuse, years of education, presence of cardiovascular disease, cognitive status at baseline and presence of endocrine disease, n=342.

| **Atrophy in 12 months**  **(volume in mm^3^)** | **Beta co-efficient** | **95%CI** | **P value** | **R^2^** |
| --- | --- | --- | --- | --- |
| Right hippocampus | -72.7 | -506.9 - 361.4 | 0.742 | 0.237 |
| Left hippocampus | -127.3 | -522.9 - 268.4 | 0.527 | 0.261 |
| Right thalamus | -429.8 | -953.2 - 93.5 | 0.107 | 0.138 |
| Left thalamus | -437.6 | -980.5 -105.2 | 0.114 | 0.096 |
| Right caudate | 25.4 | -639.1- 689.8 | 0.940 | 0.146 |
| Left caudate | 11.5 | -671.1- 694.1 | 0.974 | 0.138 |
| Right putamen | 96.6 | -90.3 - 283.5 | 0.310 | 0.161 |
| Left putamen | 298.6 | 27.4 - 569.8 | 0.031 | 0.059 |
| Right pallidum | 19.0 | -298.3 - 336.4 | 0.906 | 0.093 |
| Left pallidum | -14.3 | -574.0 - 545.3 | 0.960 | 0.076 |
| Right amygdala | -385.5 | -966.9 - 196.0 | 0.193 | 0.167 |
| Left amygdala | 264.4 | -275.9 - 804.7 | 0.336 | 0.132 |
| Right accumbens | 1098.7 | -973.4 - 3170.8 | 0.298 | 0.087 |
| Left accumbens | 1479.2 | 83.4 - 2875.0 | 0.038 | 0.094 |
| Right anterior cingulate gyrus | 86.9 | -317.0 - 490.9 | 0.672 | 0.085 |
| Left anterior cingulate gyrus | -97.5 | -474.3 - 279.3 | 0.611 | 0.119 |
| Right middle frontal gyrus | -224.9 | -592.8 – 143.0 | 0.230 | 0.130 |
| Left middle frontal gyrus | -254.7 | -607.2 - 97.9 | 0.156 | 0.124 |
| Right cuneus | -15.9 | -497.6 - 465.7 | 0.948 | 0.090 |
| Left cuneus | 104.3 | -406.6 - 615.1 | 0.688 | 0.121 |
| Right entorhinal | -163.7 | -681.3 - 353.9 | 0.534 | 0.187 |
| Left entorhinal | 3.9 | -593.4 - 601.1 | 0.990 | 0.092 |
| Right fusiform gyrus | 14.9 | -195.3 - 225.1 | 0.889 | 0.135 |
| Left fusiform gyrus | -58.8 | -322.2 - 204.6 | 0.661 | 0.133 |
| Right inferior temporal gyrus | 68.3 | -211.5 - 348.1 | 0.632 | 0.189 |
| Left inferior temporal gyrus | 144.5 | -197.1 - 486.0 | 0.406 | 0.149 |
| Right lateral orbital gyrus | -368.0 | -1121.8 - 385.8 | 0.338 | 0.054 |
| Left lateral orbital gyrus | 171.6 | -638.8 - 981.9 | 0.677 | 0.086 |
| Right lingual gyrus | 144.2 | -73.6 - 362.0 | 0.194 | 0.087 |
| Left lingual gyrus | 70.9 | -170.4 - 312.2 | 0.563 | 0.103 |
| Right medial orbital gyrus | 147.9 | -355.2 - 650.9 | 0.563 | 0.081 |
| Left medial orbital gyrus | 295.8 | -200.6 - 792.1 | 0.242 | 0.074 |
| Right middle temporal | -238.4 | -558.4 - 81.6 | 0.144 | 0.282 |
| Left middle temporal | 106.3 | -193.2 - 405.9 | 0.485 | 0.272 |
| Right pars opercularis | -324.9 | -824.8 - 175.0 | 0.202 | 0.104 |
| Left pars opercularis | -18.5 | -488.4 - 451.4 | 0.938 | 0.085 |
| Right pars orbitalis | -90.9 | -674.7 - 492.9 | 0.760 | 0.131 |
| Left pars orbitalis | -26.6 | -649.1 - 595.8 | 0.933 | 0.063 |
| Right pars triangularis | -17.3 | -488.4 - 453.9 | 0.943 | 0.154 |
| Left pars triangularis | -234.2 | -690.5 - 222.0 | 0.313 | 0.119 |
| Right postcentral gyrus medial segment | 414.1 | -645.6 -1473.9 | 0.443 | 0.093 |
| Left postcentral gyrus medial segment | 62.7 | -881.7 - 1007.1 | 0.896 | 0.081 |
| Right posterior cingulate gyrus | -85.3 | -325.1 - 154.6 | 0.485 | 0.124 |
| Left posterior cingulate gyrus | -90.6 | -307.1 - 125.8 | 0.411 | 0.110 |
| Right precentral gyrus medial segment | -362.9 | -803.8 - 78.1 | 0.106 | 0.117 |
| Left precentral gyrus medial segment | -109.2 | -524.4 - 306.0 | 0.605 | 0.069 |
| Right precuneus | -171.1 | -475.9 -133.7 | 0.270 | 0.121 |
| Left precuneus | -113.2 | -413.4 - 186.9 | 0.458 | 0.101 |
| Right superior frontal gyrus | -276.8 | -647.9 - 94.3 | 0.143 | 0.141 |
| Left superior frontal gyrus | -279.0 | -664.7 - 106.7 | 0.156 | 0.135 |
| Right superior temporal gyrus | -360.7 | -680.2 - -41.1 | 0.027 | 0.083 |
| Left superior temporal gyrus | -69.7 | -402.4 - 262.9 | 0.680 | 0.088 |
| Right anterior insula | -120.7 | -429.5 - 188.0 | 0.442 | 0.146 |
| Left anterior insula | 21.4 | -300.5 - 343.3 | 0.896 | 0.137 |
| Right posterior insula | -74.9 | -450.0 - 300.3 | 0.695 | 0.125 |
| Left posterior insula | -214.0 | -631.6 - 203.7 | 0.314 | 0.071 |

#### Table s6: Secondary analysis of the effect of PRS-D on regional brain atrophy at 12 months in the ADNI cohort, controlling for APOE genotype.

In this analysis all individuals in ADNI with a PRS-D and MRI data were included, regardless of cognitive status. Note all analyses adjusted for sex, first 4 principal components from the population stratification analysis, number of APOE ε4 alleles, baseline age, marital status, smoking status, lifetime history of alcohol abuse, years of education, presence of cardiovascular disease, cognitive status at baseline and presence of endocrine disease, n=342.

| **Atrophy in 12 months**  **(volume in mm^3^)** | **Beta co-efficient** | **95%CI** | **P value** | **R^2^** |
| --- | --- | --- | --- | --- |
| Right hippocampus | -47.4 | -478.3 - 383.5 | 0.829 | 0.260 |
| Left hippocampus | -113.8 | -506.3 - 278.7 | 0.569 | 0.284 |
| Right thalamus | -416.0 | -941.2 - 109.2 | 0.120 | 0.146 |
| Left thalamus | -411.7 | -952.4 - 128.9 | 0.135 | 0.118 |
| Right caudate | 4.7 | -663.6 - 673.0 | 0.989 | 0.150 |
| Left caudate | 30.6 | -656.4 - 717.5 | 0.930 | 0.141 |
| Right putamen | 97.7 | -89.6 - 285.1 | 0.306 | 0.171 |
| Left putamen | 312.5 | 44.1 - 581.1 | 0.023 | 0.092 |
| Right pallidum | 46.1 | -272.2 - 364.4 | 0.776 | 0.102 |
| Left pallidum | -49.3 | -612.1 - 513.5 | 0.863 | 0.081 |
| Right amygdala | -333.0 | -915.4 - 249.4 | 0.261 | 0.178 |
| Left amygdala | 321.9 | -214.6 - 858.4 | 0.239 | 0.158 |
| Right accumbens | 1144.9 | -934.3 - 3224.1 | 0.279 | 0.096 |
| Left accumbens | 1475.7 | 83.6 - 2867.8 | 0.038 | 0.114 |
| Right anterior cingulate gyrus | 64.6 | -339.9 - 469.1 | 0.754 | 0.098 |
| Left anterior cingulate gyrus | -99.6 | -477.8 - 278.6 | 0.605 | 0.127 |
| Right middle frontal gyrus | -204.2 | -568.6 -160.2 | 0.271 | 0.161 |
| Left middle frontal gyrus | -236.1 | -587.0 - 114.9 | 0.187 | 0.146 |
| Right cuneus | -14.5 | -499.1 - 470.1 | 0.953 | 0.094 |
| Left cuneus | 106.5 | -408.3 - 621.4 | 0.684 | 0.122 |
| Right entorhinal | -131.1 | -651.4 - 389.2 | 0.620 | 0.192 |
| Left entorhinal | 45.0 | -552.7 - 642.7 | 0.882 | 0.105 |
| Right fusiform gyrus | 5.2 | -206.1 - 216.5 | 0.962 | 0.140 |
| Left fusiform gyrus | -71.6 | -336.0 - 192.7 | 0.594 | 0.141 |
| Right inferior temporal gyrus | 73.6 | -208.4 - 355.6 | 0.608 | 0.190 |
| Left inferior temporal gyrus | 141.8 | -202.6 - 486.1 | 0.418 | 0.149 |
| Right lateral orbital gyrus | -261.8 | -1010.2 - 486.6 | 0.492 | 0.083 |
| Left lateral orbital gyrus | 196.6 | -613.7 - 1006.8 | 0.633 | 0.101 |
| Right lingual gyrus | 136.7 | -82.7 - 356.1 | 0.221 | 0.088 |
| Left lingual gyrus | 57.3 | -185.1 - 299.7 | 0.642 | 0.109 |
| Right medial orbital gyrus | 181.0 | -324.3 - 686.4 | 0.481 | 0.087 |
| Left medial orbital gyrus | 329.5 | -169.5 - 828.5 | 0.195 | 0.079 |
| Right middle temporal | -215.9 | -530.6 - 98.7 | 0.178 | 0.317 |
| Left middle temporal | 109.7 | -191.5 - 410.8 | 0.474 | 0.276 |
| Right pars opercularis | -302.3 | -802.1 - 197.5 | 0.235 | 0.119 |
| Left pars opercularis | -33.9 | -505.4 - 437.6 | 0.888 | 0.094 |
| Right pars orbitalis | -67.3 | -654.5 - 519.9 | 0.822 | 0.135 |
| Left pars orbitalis | -22.5 | -644.2 - 599.1 | 0.943 | 0.081 |
| Right pars triangularis | 7.2 | -465.1 - 479.4 | 0.976 | 0.164 |
| Left pars triangularis | -204.7 | -662.2 - 252.8 | 0.379 | 0.129 |
| Right postcentral gyrus medial segment | 385.7 | -680.2 - 1451.7 | 0.477 | 0.097 |
| Left postcentral gyrus medial segment | 37.6 | -910.0 - 985.2 | 0.938 | 0.090 |
| Right posterior cingulate gyrus | -99.0 | -339.7 - 141.7 | 0.419 | 0.132 |
| Left posterior cingulate gyrus | -87.8 | -304.6 - 129.0 | 0.426 | 0.122 |
| Right precentral gyrus medial segment | -376.7 | -817.7 - 64.2 | 0.094 | 0.131 |
| Left precentral gyrus medial segment | -102.9 | -518.4 - 312.6 | 0.626 | 0.083 |
| Right precuneus | -188.7 | -494.6 - 117.3 | 0.226 | 0.129 |
| Left precuneus | -111.6 | -413.9 - 190.7 | 0.468 | 0.103 |
| Right superior frontal gyrus | -261.5 | -633.9 - 110.9 | 0.168 | 0.149 |
| Left superior frontal gyrus | -262.3 | -646.7 - 122.1 | 0.180 | 0.155 |
| Right superior temporal gyrus | -366.4 | -688.4 - -44.5 | 0.026 | 0.084 |
| Left superior temporal gyrus | -82.0 | -416.7 - 252.7 | 0.630 | 0.092 |
| Right anterior insula | -110.7 | -419.7 -198.4 | 0.482 | 0.159 |
| Left anterior insula | 19.5 | -304.0 – 343.0 | 0.906 | 0.142 |
| Right posterior insula | -69.0 | -445.6 - 307.8 | 0.719 | 0.132 |
| Left posterior insula | -242.0 | -661.7 - 177.7 | 0.257 | 0.077 |

Table s7: Secondary analysis of the effect of PRS-D on regional brain volumes (mm3) at baseline in individuals without dementia at baseline in the ADNI cohort. In this analysis individuals with dementia at baseline were excluded from the analysis. Note all analyses adjusted for sex, first 4 principal components from the population stratification analysis, baseline age, marital status, smoking status, number of *APOE* ε4 alleles, lifetime history of alcohol abuse, years of education, presence of cardiovascular disease and presence of endocrine disease, n=317.

| **Brain Area**  **(total volume in mm^3^)** | **Beta co-efficient** | **95%CI** | **P value** | **R^2^** |
| --- | --- | --- | --- | --- |
| Right hippocampus | -6609.8 | -71423.4 - 58203.8 | 0.841 | 0.264 |
| Left hippocampus | -26098.4 | -85940.5 - 33743.7 | 0.391 | 0.260 |
| Right thalamus | 20075.4 | -57112.1 - 97262.8 | 0.609 | 0.263 |
| Left thalamus | 17043.2 | -63690.7 - 97777.1 | 0.678 | 0.288 |
| Right caudate | -16292.1 | -77603.4 - 45019.2 | 0.601 | 0.109 |
| Left caudate | -815.1 | -54368.1 - 52737.9 | 0.976 | 0.119 |
| Right putamen | -15461.6 | -72708.4 - 41785.3 | 0.595 | 0.197 |
| Left putamen | -21329.5 | -78968.4 - 36309.4 | 0.467 | 0.200 |
| Right pallidum | 6779.7 | -11991.9 - 25551.3 | 0.478 | 0.204 |
| Left pallidum | 2388.2 | -16667.7 - 21444.2 | 0.805 | 0.217 |
| Right amygdala | 5545.4 | -13589.8 - 24680.5 | 0.569 | 0.251 |
| Left amygdala | 3407.6 | -15063.7 – 21879.0 | 0.717 | 0.276 |
| Right accumbens | -5835.5 | -14426.9 - 2755.9 | 0.182 | 0.155 |
| Left accumbens | -4017.5 | -14081.9 - 6046.9 | 0.432 | 0.153 |
| Right ventral dc | 8870.1 | -42221.4 - 59961.5 | 0.733 | 0.335 |
| Left ventral dc | -4425.5 | -57945.5 - 49094.5 | 0.871 | 0.341 |
| Right anterior cingulate gyrus | -16892.2 | -105517.4 - 71732.9 | 0.708 | 0.123 |
| Left anterior cingulate gyrus | -31373.4 | -12818.0 - 65440.2 | 0.524 | 0.173 |
| Right middle frontal gyrus | 8596.1 | 260132.9 - 277325.2 | 0.950 | 0.232 |
| Left middle frontal gyrus | -11612.4 | -281524.1 - 258299.3 | 0.933 | 0.201 |
| Right cuneus | -35626.8 | -137737.5 - 66483.9 | 0.493 | 0.181 |
| Left cuneus | -84251.1 | -176482.8 7980.5 | 0.073 | 0.223 |
| Right entorhinal | 4275.2 | -42874.9 - 51425.2 | 0.858 | 0.344 |
| Left entorhinal | -12948.5 | -58332.2 - 32435.3 | 0.575 | 0.308 |
| Right fusiform gyrus | 7936.0 | -119054.1 - 134926.2 | 0.902 | 0.360 |
| Left fusiform gyrus | -26275.6 | -142481.6 - 89930.5 | 0.657 | 0.361 |
| Right inferior temporal gyrus | -69980.7 | -252228.0 - 112266.6 | 0.450 | 0.309 |
| Left inferior temporal gyrus | -35638.4 | -211532.6 -140255.8 | 0.690 | 0.323 |
| Right lateral orbital gyrus | 18944.4 | -31514.7 - 69403.6 | 0.461 | 0.148 |
| Left lateral orbital gyrus | 13114.8 | -39427.5 - 65657.0 | 0.624 | 0.133 |
| Right lingual gyrus | -26072.7 | -167918.2 - 115772.8 | 0.718 | 0.223 |
| Left lingual gyrus | 18944.4 | -31514.7 - 69403.6 | 0.461 | 0.148 |
| Right medial orbitofrontal | -19346.0 | -79730.4 - 41038.4 | 0.529 | 0.266 |
| Left medial orbitofrontal | -26053.6 | -103791.1 - 51684.0 | 0.510 | 0.193 |
| Right middle temporal | 47962.7 | -189415.8 - 285341.1 | 0.691 | 0.220 |
| Left middle temporal | 85644.3 | -145674.2 - 316962.8 | 0.467 | 0.258 |
| Right parahippocampal gyrus | -9828.8 | -57721.9 - 38064.4 | 0.687 | 0.356 |
| Left parahippocampal gyrus | -5368.1 | -55074.6 - 44338.4 | 0.832 | 0.358 |
| Right pars opercularis | 58801.1 | -20108.0 - 137710.2 | 0.144 | 0.158 |
| Left pars opercularis | 60845.4 | -12078.3 -133769.2 | 0.102 | 0.203 |
| Right pars orbitalis | 5670.7 | -40142.9 - 51484.4 | 0.808 | 0.178 |
| Left pars orbitalis | -2792.3 | -48045.6 - 42460.9 | 0.903 | 0.189 |
| Right pars triangularis | 73588.2 | 6888.5 - 140287.8 | 0.031 | 0.133 |
| Left pars triangularis | 31959.8 | -34042.3 - 97961.8 | 0.341 | 0.169 |
| Right calcarine cortex | -8491.7 | -80070.0 - 63086.6 | 0.816 | 0.136 |
| Left calcarine cortex | -22207.7 | -9834.0 - 53930.7 | 0.566 | 0.155 |
| Right postcentral gyrus | -51584.3 | -209351.3 - 106182.7 | 0.520 | 0.141 |
| Left postcentral gyrus | -34045.2 | -198679.3 - 130588.9 | 0.684 | 0.180 |
| Right posterior cingulate gyrus | -1862.5 | -71659.9 - 67934.9 | 0.958 | 0.210 |
| Left posterior cingulate gyrus | 21809.9 | -46514.4 - 90134.3 | 0.530 | 0.280 |
| Right precentral | -125257.0 | -308929.6 - 58415.6 | 0.181 | 0.262 |
| Left precentral | -19154.1 | -210838.2 -172530.1 | 0.844 | 0.224 |
| Right precuneus | 65110.2 | -127364.0 - 257584.5 | 0.506 | 0.228 |
| Left precuneus | 79133.7 | -109700.4 - 267967.8 | 0.410 | 0.251 |
| Right superior frontal gyrus | 74518.3 | -116919.3 – 265956.0 | 0.444 | 0.217 |
| Left superior frontal gyrus | 16481.9 | -168604.3 – 201568.0 | 0.861 | 0.236 |
| Right superior parietal | 122460.6 | -34918.1 - 279839.3 | 0.127 | 0.212 |
| Left superior parietal | 109862.7 | -60461.6 - 280187.0 | 0.205 | 0.205 |
| Right superior temporal gyrus | 33856.1 | -87873.4 -155585.6 | 0.585 | 0.184 |
| Left superior temporal gyrus | -30090.0 | -159654.4 - 99474.5 | 0.648 | 0.201 |
| Right anterior insula | -7342.0 | -75812.4 - 61128.4 | 0.833 | 0.202 |
| Left anterior insula | -3667.7 | -69281.7 - 61946.4 | 0.912 | 0.223 |
| Right posterior insula | 27628.8 | -14426.6 - 69684.1 | 0.197 | 0.232 |
| Left posterior insula | 24415.8 | -16293.2 - 65124.8 | 0.239 | 0.232 |
| Right transverse temporal | -13943.0 | -52764.9 - 24879.0 | 0.480 | 0.207 |
| Left transverse temporal | 30558.3 | -15873.3 - 76989.8 | 0.196 | 0.188 |
